# Inhibition of AKT-Signaling Sensitizes Soft Tissue Sarcomas (STS) and Gastrointestinal Stromal Tumors (GIST) to Doxorubicin via Targeting of Homology-Mediated DNA Repair

**DOI:** 10.3390/ijms21228842

**Published:** 2020-11-22

**Authors:** Sergei Boichuk, Firuza Bikinieva, Ilmira Nurgatina, Pavel Dunaev, Elena Valeeva, Aida Aukhadieva, Alexey Sabirov, Aigul Galembikova

**Affiliations:** 1 Department of Pathology, Kazan State Medical University, 420012 Kazan, Russia; firuza1995@mail.ru (F.B.); ilmiranurgatina@gmail.com (I.N.); dunaevpavel@mail.ru (P.D.); arom1705@mail.ru (A.A.); ailuk000@mail.ru (A.G.); 2 Central Research Laboratory, Kazan State Medical University, 420012 Kazan, Russia; vevaleeva@ya.ru; 3 Department of Pathology, Tatarstan Cancer Center, 420029 Kazan, Russia; a-sabirov@yandex.ru

**Keywords:** soft tissue sarcomas (STS), gastrointestinal stromal tumors (GIST), AKT signaling, Rad51 recombinase, homology-mediated DNA repair, apoptosis, sensitization

## Abstract

Activation of the phosphoinositide 3-kinase (PI3K)/Akt/mTOR pathway is well documented for a broad spectrum of human malignancies supporting their growth and progression. Accumulating evidence has also implicated AKT as a potent modulator of anti-cancer therapies via regulation of DNA damage response and repair (DDR) induced by certain chemotherapeutic agents and ionizing radiation (IR). In the present study, we examined the role of AKT signaling in regulating of Rad51 turnover and cytotoxic effects of topoisomerase II inhibitor, doxorubicin (Dox) in soft tissue sarcomas (STS) and gastrointestinal stromal tumors (GIST) in vitro. Blocking of AKT signaling (MK-2206) enhanced cytotoxic and pro-apoptotic effects of Dox in vast majority of STS and GIST cell lines. The phosphorylated form of Akt co-immunoprecipitates with Rad51 after Dox-induced DNA damage, whereas Akt inhibition interrupts this interaction and decreases Rad51 protein level by enhancing protein instability via proteasome-dependent degradation. Inhibition of Akt signaling in Dox-treated cells was associated with the increased number of γ-H2AX-positive cells, decrease of Rad51 foci formation and its colocalization with γ-H2AX foci, thereby revealing unsuccessful DDR events. This was also in consistency with an increase of tail moment (TM) and olive tail moment (OTM) in Dox-treated GIST and STS cells cultured in presence of Akt inhibitor after Dox washout. Altogether, our data illustrates that inhibition of AKT signaling is STS and GIST might potentiate the cytotoxic effect of topoisomerase II inhibitors via attenuating the homology-mediated DNA repair.

## 1. Introduction

Activation of the phosphoinositide 3-kinase (PI3K)/Akt/mTOR pathway is frequently detected in human malignancies, including gastrointestinal stromal tumors (GIST) and soft tissue sarcomas (STS). This pathway supports tumor growth and progression and is thought to be linked with resistance to the current therapeutic regimens, including certain chemotherapeutic agents, ionizing radiation (IR) and targeted-based therapies. In particular, activation of PI3K/Akt/mTOR pathway in GIST is due to the constitutive activation of c-KIT/PDGFRA autophosphorylation and involved in oncogenesis and tumor progression at various disease stages [1], and is also thought to be linked with GIST resistance to imatinib mesylate (IM) [2,3]. Moreover, the preclinical experiments illustrated that a long-term exposure of IM- and sunitinib (SU)-resistant cell lines to SU induces epigenetic silencing of *PTEN* with a consequent overactivation of the PI3K/AKT pathway [4]. This also correlates with a clinical data illustrating that low/negative IHC-staining for PTEN was associated with aggressive disease [5], thereby suggesting that *PTEN* depletion and/or silencing is also associated with aggressive phenotype and resistance to RTK inhibition. Important, molecular and genomic changes in IM-resistant GISTs illustrated that the PI3K/Akt/mTOR pathway has a greater importance in IM-resistant GIST than other pathways downstream of c-KIT or PDGFRA (e.g., MEK/MAPK pathway), therefore illustrating a rationale for targeting the PI3K/Akt/mTOR pathway in GIST [6]. Indeed, inhibition of PI3K [7], AKT [8] and mTOR [1] has been shown promising results in vitro and in xenograft models and led to the clinical trials to examine an efficiency against IM-resistant GIST. However, some of them have not succeeded to date [9,10], whereas the other trials to examine the novel drugs targeting the elements of this pathway are currently ongoing (NCT01991379, NCT01735968 and NCT01468688).

Activation of the PI3K/AKT/mTOR pathway is also well-documented for soft tissue sarcomas (STS). In particular, for leiomyosarcoma (LMS) the most common genetic abnormalities include the loss of function mutations in *p53* and/or *PTEN* or activating mutations in the genes encoding the signaling molecules of the PI3K/AKT/mTOR pathway [11,12,13]. Similarly, to LMS, the PI3K/AKT/mTOR signaling pathway is often aberrantly activated in Ewing’s sarcoma (ES) [14,15], rhabdomyosarcoma, a well-known pediatric sarcoma of soft tissues displaying very similar histology and therapeutic options with ES [16], osteosarcoma (OS) [17,18], thereby illustrating that the PI3K/AKT/mTOR pathway is a suitable therapeutic target for STS as well as for other human cancers.

Besides, the PI3K/AKT/mTOR pathway is considered as a perspective molecular therapeutic target for STS and GIST, accumulating evidence also illustrates the tight connection between this pathway and checkpoint responses and repair of DNA damage, induced by certain chemotherapeutic agents and/or ionizing radiation. This reflects, predominantly, the regulatory role of AKT in DNA double-stand break (DSB) repair, including non-homology end-joining (NHEJ) and homologous recombination (HR)—mediated DNA repair, which in turn also illustrates the AKT-mediated pathway, a perspective target to sensitize STS and GIST to DNA-damaging agents, including topoisomerase II (Topo II) inhibitors. Given that Topo II inhibitors are currently used for therapy of STS [19,20,21,22] and taking into account that GIST were also previously shown to be sensitive to the Topo II inhibitors [23,24], we thought to examine whether inhibition of the PI3K/AKT/mTOR pathway might enhance their sensitivity to Topo II inhibitor, doxorubicin (DOX) via targeting of the molecular pathways involved in DNA DSB repair.

In the present study we characterized the importance of AKT-pathway for HR-mediated repair of DSBs in STS and GIST in vitro and their relevance to the tumor cell sensitivity to topoisomerase II inhibitor, doxorubicin (Dox). We found that inhibition of AKT-signaling in GIST and STS cell lines results in a significantly decreased expression of Rad51 recombinase and number of residual Rad51/BRCA1 foci in Dox-treated tumor cells. This was due to the decreased stability of Rad51 as a consequence of the enhanced proteasomal degradation. Finally, as a result of the impaired homology-mediated DNA repair, we observed a substantial decrease of viability in AKT-inhibited tumor cells after Dox treatment, which was evidenced by MTS-based colorimetric assay and increased expression of apoptotic markers (cleaved forms of caspase-3 and poly-(ADP)-ribose-polymerase (PARP) and the numbers of hypodiploid cells).

Therefore, overactivation of AKT-signaling pathway in STS and GIST might serve as a prospective molecular target to enhance cytotoxic effects of DNA-topoisomerase II inhibitors inducing DNA DSBs in STS and GIST.

## 2. Results

### 2.1. Inhibition of AKT-Signaling Enhances Cytotoxicity of Topo II Inhibitors in STS and GIST

**Figure 1:**
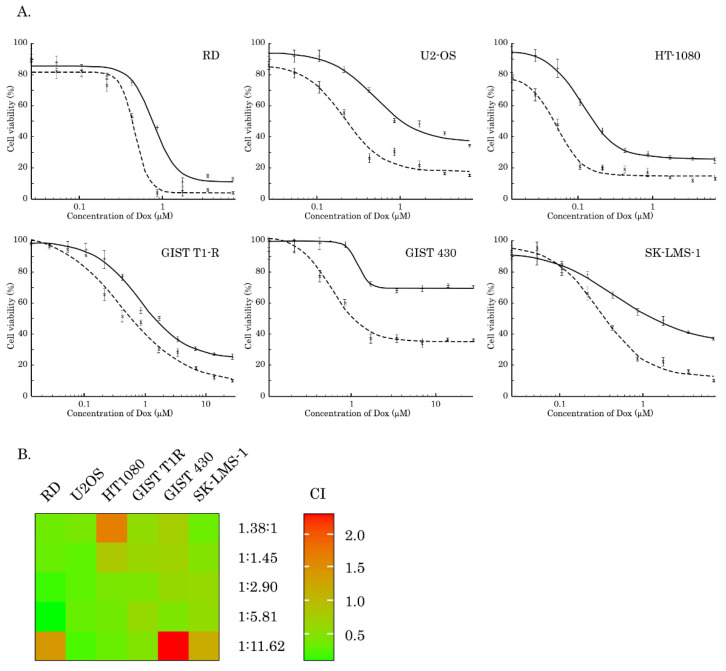
An impact of AKT inhibition on cytotoxic activity of doxorubicin (Dox) in soft tissue sarcomas (STS) and gastrointestinal stromal tumors (GIST). (**A**) MTS-based viability assay in RD rhabdomyosarcoma, U2-OS osteosarcoma, HT-1080 fibrosarcoma, GIST T-1R, GIST 430 and SK-LMS-1 leiomyosarcoma cells treated with Dox alone (solid lines) or in combination of MK-2206 (dashed lines) for 72 h. The data was normalized to DMSO-treated controls. Values are the means ± standard deviation (*n* = 3). (**B**) the heat-map illustrating the combination index (CI) values for Dox and MK2206 combinations at various molar ratios for cancer cell lines indicated above. CI values were calculated with CompuSyn Software (Version 1.0), based on the Chou–Talalay algorithm. CI values < 1 indicate the synergy between Dox and MK-2206.

To examine whether inhibition of AKT signaling potentiates the cytotoxic activities of Dox in STS and GIST, we performed MTS-based survival assay with a broad spectrum of cancer cell lines, including SK-LMS-1 leiomyosarcoma, RD rhabdomyosarcoma, HT-1080 fibrosarcoma, A673 Ewing’s sarcoma, U2-OS osteosarcoma, IM-sensitive and resistant gastrointestinal stromal tumors (e.g., GIST T-1 vs. GIST T-1R and GIST 430, respectively). Cells indicated above were treated with Dox for 72 h alone or in combination with MK-2206, a selective AKT-inhibitor. We observed that inhibition of AKT signaling potentiated cytotoxic activity of Dox and inhibited growth of the vast majority of cancer cell lines included in present study (Figure 1). The IC50 values for MK-2206, Dox used alone or in combination are shown on Table 1. In particular, we observed an approximately two-fold decrease of IC50 for all types of cancer cell lines treated with combination of Dox and MK-2206, when compared to cells treated with Dox alone. Synergism of Dox and MK-2206 was also calculated by combination index (CI) values for each molar ratio of Dox and MK-2206 (Table 2) and was depicted as a heat-map shown on Figure 1B. In addition, the average synergistic effects of Dox and MK-2206 were calculated by using of R-package of computational tool Synergy Finder. Indeed, we observed a prominent synergism for Dox and MK-2206 in RD and SK-LMS-1 cells (Figure S1) and other STS cell lines, including U2-OS osteosarcoma and HT-1080 fibrosarcoma cells (Figure S2).

### 2.2. Inhibition of AKT-Signaling Enhances Doxorubicin-Induced Apoptosis of STS and GIST

**Figure 2:**
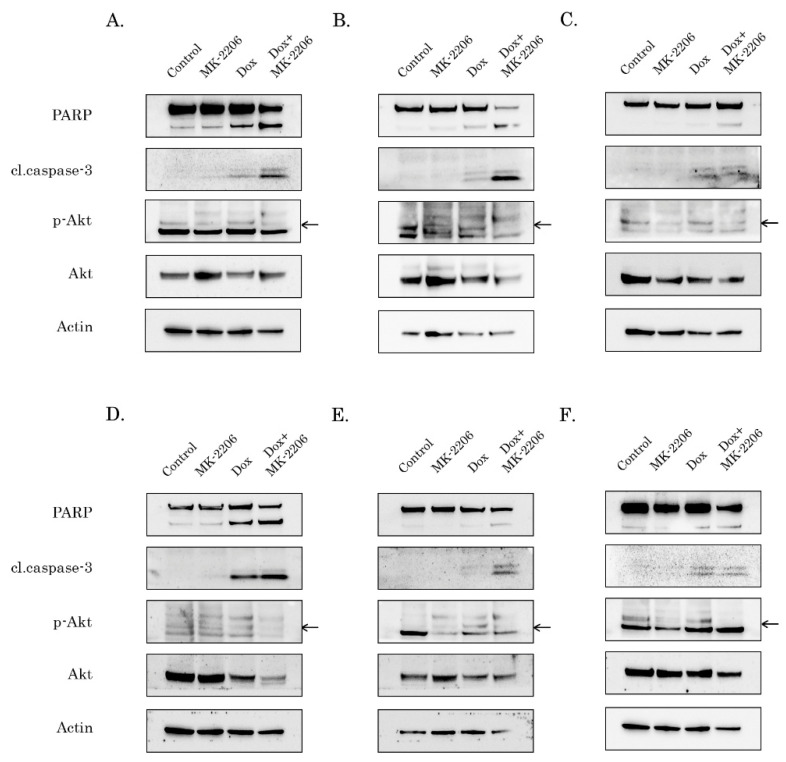
AKT inhibition potentiates pro-apoptotic activity of Dox in STS and GIST. Immunoblot analysis for apoptosis markers (cleaved forms of poly-(ADP)-ribose-polymerase (PARP) and caspase-3) in RD rhabdomyosarcoma (**A**), U2-OS osteosarcoma (**B**), HT-1080 fibrosarcoma (**C**), GIST T-1R (**D**), GIST 430 (**E**) and SK-LMS-1 leiomyosarcoma (**F**) cells after treatment with DMSO (control), Dox (0.25 g/mL) and MK-2206 (5 M) alone and in combination (e.g., Dox + MK-2206) for 72 h. The lysates were also probed for total and phosphorylated forms of AKT to illustrate AKT inhibition by MK-2206. pAKT expression is shown by arrows. Actin was used as a loading control.

To further corroborate these findings, we examined whether inhibition of AKT signaling can potentiate pro-apoptotic effect of Dox in STS and GIST. For this purpose, cancer cell lines were treated with the low dose of Dox (0.25 g/mL) in absence (control) or presence of MK-2206 (5 M) and further subjected for western blotting to examine expression of apoptotic markers (cleaved forms of PARP and caspase-3). Strikingly, a substantial increase of cleaved caspase-3 and PARP was observed in the majority of STS and GIST cells treated with combination of AKT and Topo II inhibitors (Figure 2). As expected, MK-2206 used alone has no cytotoxic effect on the tumor cells, whereas pro-apoptotic effect of Dox used alone was much less when compared to the effect of combination of these inhibitors. The most sensitive for AKT inhibition were Dox-treated RD rhabdomyosarcoma cells (Figure 2A), U2-OS osteosarcoma (Figure 2B), GIST T-1R (Figure 2D) and 430 (Figure 2E) which was evidenced by a substantial cell death after the treatment with Dox and MK-2206. Quantification by mean pixel density revealed that PARP and caspase-3 cleavage was substantially increased in all types of cancer cell lines treated with Dox in combination of MK-2206 (Figure S3.1). The inhibitory effect of MK-2206 on AKT pathway in tumor cells was confirmed by a substantial decrease of AKT phosphorylation (Figure 2). Similarly, changes in phospho-AKT (Ser473) expression were quantified and normalized to actin (Figure S3.1), thereby illustrating that decreased AKT phosphorylation in MK-2206-inhibited cells was specific and was not due to the changes in expression of the total AKT. 

In addition to increased levels of cleaved caspase-3 and PARP in cancer cells treated with Dox in presence of AKT inhibitor, we also observed the increased numbers of apoptotic (e.g., Annexin V-positive cells) cells in RD rhabdomyosarcoma and GIST T-1R cells treated with combination of Dox and MK-2206 when compared to non-treated cells or cells treated with Dox alone (Figure S4), thereby revealing that AKT inhibition effectively sensitized STS and GIST cells to Dox treatment and induced apoptotic cell death. 

### 2.3. Inhibition of AKT Signaling Attenuates Repair of Dox-Induced DNA Damage

**Figure 3:**
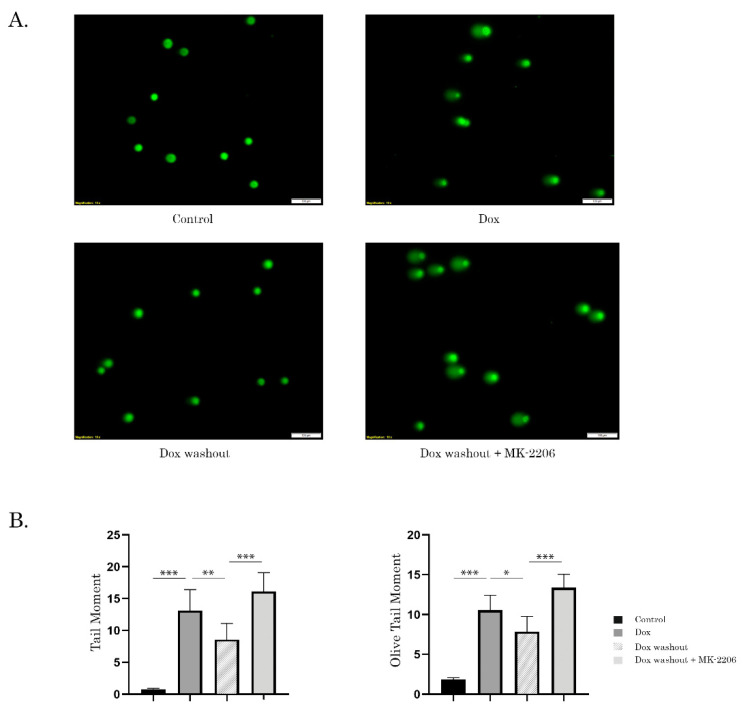
Inhibition of AKT-signaling attenuates DNA double-stand break (DSB) repair. GIST T-1R cells treated with DMSO (negative control) and Dox 0.5 μg/mL for 2 h. After the drugs were washed out, cancer cells were further cultured in absence (Dox washout) or presence of MK-2206 (5 µM), an AKT inhibitor (Dox washout + MK-2206) for 8 h. (**A**) representative images of comets from the experimental settings shown above (Scale bars = 100 μm). (**B**) graphic depiction of the calculated tail moment (TM) and olive tail moment (OTM) from alkaline comet assay shown in Figure 3A. Columns, mean of at least three independent experiments with a minimum of 50 cells counted per each experiment; bars, SE. * *p* < 0.05; ** *p* < 0.01; *** *p* < 0.001.

Next, we performed alkaline-based DNA comet assay to examine whether enhanced apoptosis of cancer cells treated with Dox and MK-2206 was due to DNA damage which remained unrepairable in presence of AKT inhibitor. For this purpose, STS or GIST cells were initially treated with Dox for 2 h to induce DNA damage, and followed by the wash-out of chemotherapeutic agent and further cultured for 8 h in absence or presence of MK-2206. Strikingly, we observed a substantial increase of tail moment (TM) and olive tail moment (OTM) in GIST cells, thereby illustrating unrepairable DNA damage in AKT-inhibited tumor cells (Figure 3A,B). Similar data was obtained in GIST cells that were not washed from Dox. Again, we observed that MK-2206 substantially attenuated DNA damage repair which was evidenced by increased TM (Figure S5A,B). As expected, we observed a substantial increase of TM in Dox-treated cells when compared to the previous experimental settings, thereby illustrating the substantial DNA damage due to the permanent exposure to Dox. Given that alkaline-based DNA comet assay aimed to detect all the types of DNA lesions, we performed neutral version of single-cell electrophoresis to examine whether inhibition of AKT-signaling attenuates repair of Dox-induced DNA DSBs. Similar to alkaline version, we observed an increase of both TM and OTM in GIST cells treated with Dox in presence of MK-2206, thereby revealing that AKT-signaling plays an important role in regulating of repair of Dox-induced DNA lesions, including DNA DSBs (Figure S5C,D). 

**Figure 4:**
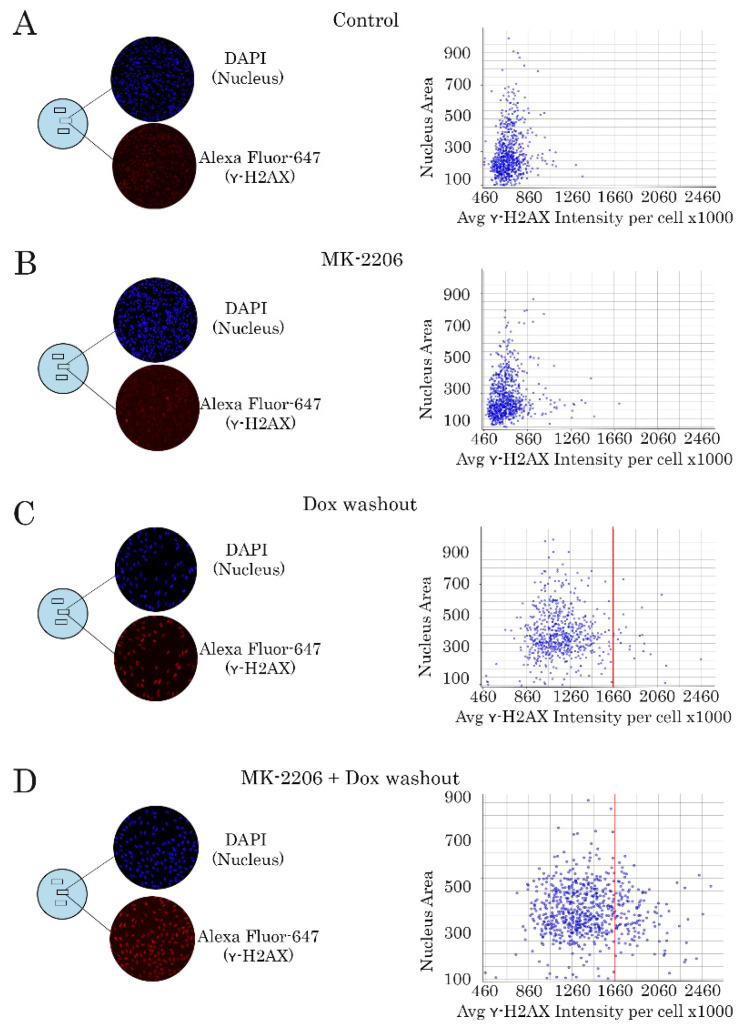
Inhibition of AKT-signaling delays the kinetics of γ-H2AX decline in Dox-treated GIST T-1R cells (representative experiment). Left panel-cancer cells were treated with DMSO (negative control) (**A**), MK-2206, a selective AKT inhibitor (5 µM) (**B**), and Dox (0.5 μg/mL) for 2 h. The cells were further washed from the compounds indicated above and cultured for 8 h in absence (Dox washout) (**C**) or presence of MK-2206 (Dox washout + MK-2206) (**D**). Right panel-histograms illustrating the intensity of γ-H2AX-specific fluorescence at the single-nucleus level. GIST T-1R cells were grown on slides for 24 h and treated with Dox and MK-2206 as indicated above. Cells were fixed with paraformaldehyde and stained with DAPI (blue) and γ-H2AX-specific antibody (red). The intensity of γ-H2AX-specific fluorescence was measured for each nucleus (DAPI) and calculated automatically. All images were acquired by GE Cytell imager as shown in Section 4.10.

**Figure 5:**
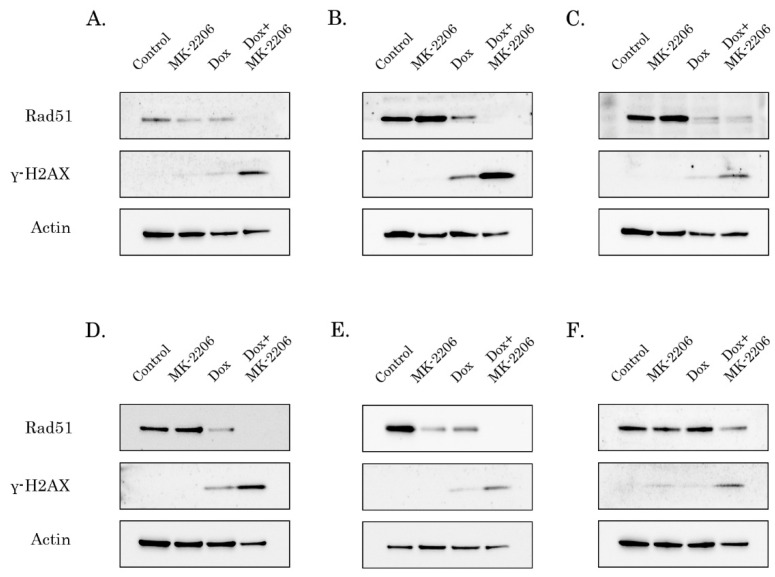
An impact of AKT inhibition on γ-H2AX and Rad51 recombinase in cancer cells. Immunoblot analysis was performed on RD rhabdomyosarcoma (**A**), U2-OS osteosarcoma (**B**), HT-1080 fibrosarcoma (**C**), GIST T-1R (**D**), GIST 430 (**E**), SK-LMS-1 leiomyosarcoma and (**F**) treated with DMSO (control), Dox (0.25 g/mL), MK-2206 (5 M) alone and in combination (e.g., Dox + MK-2206) for 48–72 h. Actin was used as a loading control.

Unrepairable DNA damage in Dox-treated tumor cells cultured in presence of AKT inhibitor was also confirmed by quantitative analysis of γ-H2AX-expression, a well-known marker of the DNA DSBs. As expected, MK-2206 used alone has no impact on γ-H2AX-expression, thereby revealing absence of DNA DSBs in GIST T-1R cells treated with AKT inhibitor (Figure 4B). In contrast, Dox-treated cells exhibited an increased pattern of γ-H2AX-expression. Of note, Dox was washed out from the cell culture 2 h post treatment to exclude permanent DNA damage, and hereby allowing to repair Dox-induced DNA lesions (Figure 4C). Strikingly, we observed a retention of γ-H2AX-expression after AKT inhibition in vast majority of Dox-treated GIST cells (Figure 4D). Similar findings were observed for various STS cell lines, including HT-1080 fibrosarcoma cells, as shown in Figure S6.1. In contrast to the AKT inhibitor, inhibition of MAPK-signaling pathway by selective inhibitor U0126, did not have the similar effects on DNA repair in Dox-treated GIST (Figure S6.2). WB data also revealed a substantial increase of γ-H2AX-expression in all tumor cell lines treated with combination of Dox and MK-2206 when compared to the cells treated with Dox alone (Figure 5). Strikingly, our WB data also illustrated a substantial decrease of Rad51 expression in most of Dox-treated cells (Figure 4). 

All together this data illustrates that inhibition of AKT-signaling in STS and GIST sensitizes them to Dox due to the inhibition of DNA damage repair.

### 2.4. Rad51 Expression Is Substantially Reduced in AKT-Inhibited Cancer Cells

**Figure 6:**
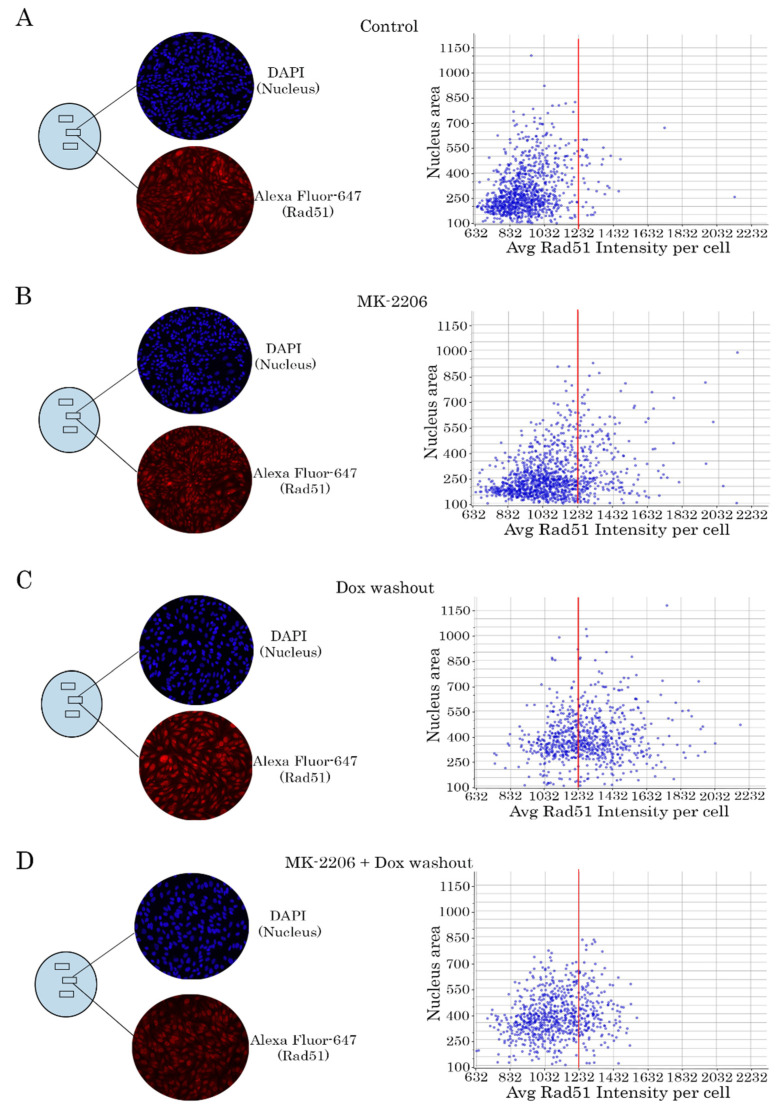
Inhibition of AKT-signaling attenuates Rad51 expression in Dox-treated cells (representative experiment). (**A**–**D**) GIST T-1R cells were treated similarly as shown for Figure 4. Left panel—cells were fixed with paraformaldehyde and stained with DAPI (blue) to outline the nucleus and Rad51-specific antibody (red). The intensity of Rad51-specific fluorescence was assayed for each nucleus (DAPI) and quantified automatically. Right panel—histograms illustrating the intensity of Rad51-specific fluorescence at the single-nucleus level. All images were acquired by GE Cytell imager as shown in “Materials and methods”.

Given that attenuation of DNA damage repair in AKT-inhibited cancer cells might be due to the decreased efficacy of homology-mediated repair of DNA DSBs, we analyzed the expression of Rad51 recombinase, known as a key protein involved in DSB repair. For this purpose, we performed a quantitative analysis of Rad51 expression in cancer cells treated with Dox and MK-2206 by a similar way as shown on Figure 4. As expected, no difference in Rad51 expression was found between control (Figure 6A) and MK-2206-treated cells (Figure 6B). When Dox-treated cells were washed out (to exclude a permanent DNA damage), we found a substantial increase of Rad51 intensity in cancer cells (Figure 6C). Strikingly, intensity of Rad51-mediated fluorescence substantially decreased when MK-2206 was introduced in cell culture after Dox washout (Figure 6D). This was observed for GIST T-1R cells (Figure 6) and STS (data not shown), thereby illustrating the possible mechanism of MK-2206-induced sensitization of cancer cells to Topo II inhibitors. These findings were in consistency with WB data illustrating a substantial decrease of Rad51 expression in AKT-inhibited cancer cells (Figure 5). 

**Figure 7:**
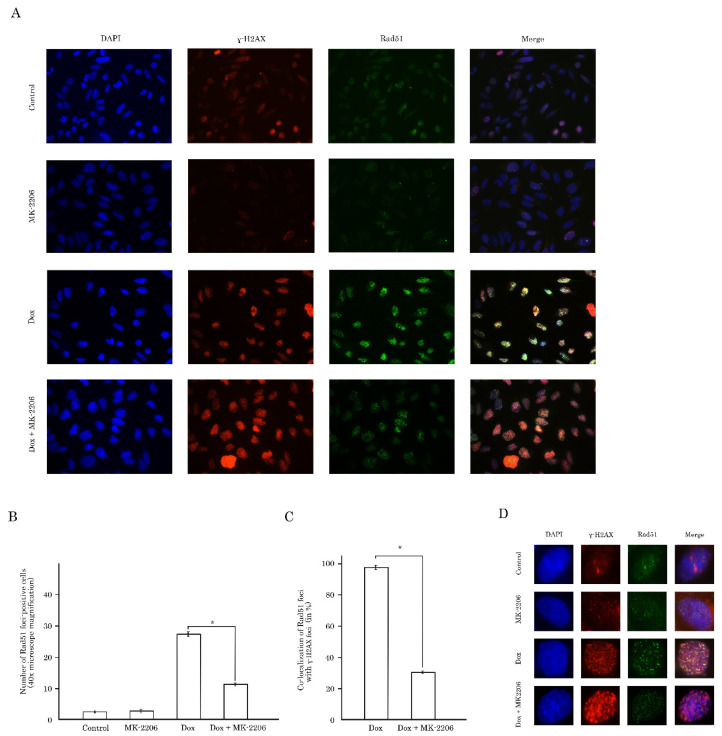
Blockage of Akt signaling pathway disrupts involvement of the Rad51 protein in the repair of DNA DSBs in GIST T-1R cells. (**A**) immunofluorescence staining of GIST T-1R cells for γ-H2AX and Rad51. The cells were cultured in the presence of DMSO (control), MK-2206 (5 µM for 48 h), Dox (0.25 µg/mL for 4 h) or pretreated with MK-2206 (5 µM) for 48 h prior Dox treatment. DAPI staining (blue) was used to outline the nucleus. Scale bars = 20 μm. (**B**) graph depicting the number of GIST T-1R cells positive for Rad51 foci after Dox treatment alone or in presence of MK-2206 from three independent experiments. Cells treated with DMSO (control) and MK-2206 were used as the negative controls. * *p* < 0.05. (**C**) graph showing co-localization of Rad51 foci with γ-H2AX foci in GIST T1-R cells after Dox treatment alone or in presence of MK-2206 from three independent experiments. * *p* < 0.05. (**D**) distribution of γ-H2AX and Rad51 foci in the nucleus in a single-cell level. GIST T-1R cells were pretreated with DMSO (control) or MK-2206 (5 µM) for 48 h prior Dox treatment (0.25 µg/mL for four hours). DAPI staining (blue) was used to outline the nucleus. Scale bars = 10 μm.

The impact of AKT-signaling on Rad51 expression was also assessed by immunofluorescence staining. Non-treated GIST T-1R cells exhibited a low level of γ-H2AX/Rad51 foci, whereas vast majority of tumor cells became γ-H2AX/Rad51-foci positive after Dox treatment (Figure 7A). 

Strikingly, a substantial decrease of Rad51 foci-positive cells was found when cancer cells were treated with Dox in combination with MK-2206 (Figure 7A,B). Moreover, inhibition of AKT-signaling attenuated co-localization between Rad51 and γ-H2AX foci after Dox treatment (Figure 7C, bottom panel), thus suggesting a failure in the recruitment of Rad51 to DSBs in AKT-inhibited tumor cells after Dox treatment. Similar results were observed for STS cells lines, including RD rhabdomyosarcoma cells, as shown in Figure S7. Indeed, the majority of Dox-treated cells exhibited high co-localization between Rad51 and γ-H2AX foci, whereas inhibition of AKT-signaling dramatically reduced the number of cells with co-localized proteins indicated above. 

Of note, increased number of Rad51 foci in Dox-treated cells was in contrast to the substantial decrease of Rad51 expression observed in most of cancer cells utilized in present study, as shown in Figure 4. These might be due to the differences in the methodologies utilized for these assays. Indeed, RIPA buffer which is commonly utilized for WB analysis might be not effective in extracting of the chromatin-bound proteins, whereas the increased number of Rad51 nuclear foci observed during immunofluorescence staining mainly represents Rad51 associated with chromatin. To confirm this possibility, we examined the distribution of Rad51 in different subcellular fractions in GIST/STS cells treated with Dox alone or in presence of AKT inhibitor, as well. The results showed that while in control cells Rad51 was evenly distributed between Triton X-100 soluble (i.e., cytoplasmic and soluble nuclear) and DNAase-soluble fractions (i.e., chromatin-associated), inhibition of AKT-signaling with MK-2206 induced its re-distribution from the chromatin, whereas Dox brought a pronounced immobilization of the protein in the DNAase-soluble fraction (Figure S8). Strikingly, inhibition of AKT signaling in Dox-treated cells attenuated recruitment of Rad51 in chromatin-associated fraction, thereby revealing the attenuation of Rad51-mediated repair mechanisms. In contrast to Rad51, expression of pRPA (Ser 4/8) in DNA-soluble fraction did not differ between GIST T-1R cells treated with Dox alone or in presence with AKT inhibitor, thereby suggesting that processing of DSBs in AKT-inhibited cells remained unchanged. 

### 2.5. AKT Impacts Rad51 Stability in GIST and STS Cell Lines

Given that AKT inhibition has a negative impact of Rad51 levels in the number of Rad51 foci in Dox-treated GIST and STS, we further examined the molecular mechanisms responsible of this phenomenon.

First, RT-PCR data illustrated very small, and statistically insignificant differences between Rad51 mRNA transcripts in control and MK-2206 treated GIST T-1R cells. Similarly, no differences in Rad51 *mRNA* levels were observed between non-treated and MK-2206-treated SK-LMS-1 leiomyosarcoma and RD rhabdomyosarcoma cell lines, thereby revealing that inhibition of AKT-signaling has no impact on *Rad51* transcription in GIST and STS (Figure S9).

**Figure 8:**
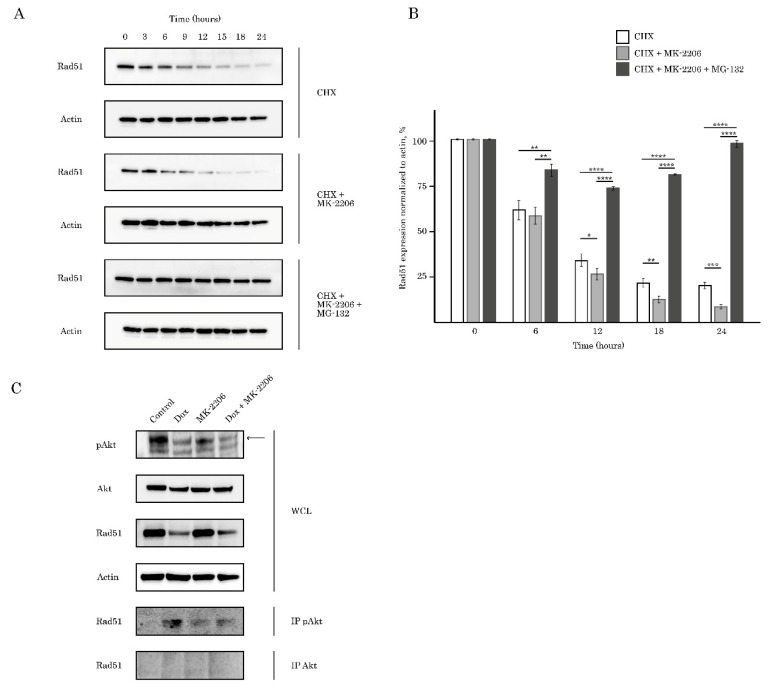
AKT is required for regulation of Rad51 stability. (**A**) the half-life of Rad51 was determined by cycloheximide (CHX) chase analysis. GIST T-1R cells were treated with MK-2206 (5 μM) or DMSO (control), followed by treatment with 10 µg/mL cycloheximide for 0, 3, 6, 9, 12, 15, 18 and 24 h. Immunoblotting for Rad51 and actin was performed on samples from all time points. To examine an impact of AKT-inhibition on proteasomal degradation of Rad51, cells were treated with MK-2206 and CHX in presence of MG-132 (2 μM) and were subjected for immunoblotting for Rad51 and actin as a loading control. (**B**) densitometric analysis of Rad51 in CHX chase analysis indicated above; bars, SD. * *p* < 0.05; ** *p* < 0.01; *** *p* < 0.001; **** *p* < 0.0001. (**C**) GIST T-1R cell lysates were immunoprecipitated with total (left) or phosphorylated at Ser473 (right) AKT Abs and immunoblotted with Rad51 Abs to demonstrate endogenous complex formation. A whole cell lysate (WCL) was included. pAKT expression is shown by arrows. Actin was used as a loading control. For immunoprecipitation experiments cells were treated with Dox (1 μg/mL) and MK-2206 (10 μM).

Next, we examined whether low levels of Rad51 in AKT-inhibited cells were due to increased turnover of the protein via ubiquitin-mediated proteasomal pathway. To test this possibility, we initially inhibited synthesis of the new protein by cycloheximide (CHX) and compared Rad51 levels in control and MK-2206-treated cells. Indeed, we observed the substantial decrease of half-life of Rad51 in GIST cells treated with CHX in presence of MK-2206, thereby suggesting about the rapid turnover of protein in AKT-inhibited cancer cells (Figure 8, upper and middle panels). Next, we found that MG-132, a 26S proteasome inhibitor effectively restored Rad51 levels in AKT-inhibited cells, thereby revealing an increase proteasome-mediated degradation of Rad51 in MK-2006-treated cells (Figure 8, bottom panel).

Given the functional link between Akt and Rad51, we hypothesized that they might be in a complex together. For this purpose, we examined the interaction between endogenous Akt/pAkt and Rad51. Lysates from GIST T-1R cells were immunoprecipitated for Akt or pAkt Ser473, followed by Western blotting with Rad51 antibody to detect the complexes. As shown in Figure 8B, specific co-immunoprecipitation was detected between Rad51 and pAkt (but not total Akt) after Dox treatment, thus confirming the interaction with endogenous proteins and reveling physical interaction between activated Akt and Rad51 after DNA damage. Strikingly, inhibition of AKT signaling in Dox-treated cells abolished this interaction. As expected, negative results were also observed in control (non-treated cells) and cells exposed to MK-2206.

All together, these results suggest that AKT inhibition in tumor cells leads to Rad51 down-regulation via ubiquitin-mediated proteasome pathway.

Collectively, our data illustrates that inhibition of AKT pathway in GIST and STS attenuates homology-mediated DNA repair and sensitizes tumor cells to the DNA-damaging agents, such as doxorubicin.

## 3. Discussion

It is well-known that an efficiency of most commonly used anticancer agents, including the classic genotoxic chemotherapeutic drugs and ionizing radiation, is due to their DNA-damaging properties which in turn triggers apoptotic cell death of cancer cells harboring unrepairable DNA lesions. Apoptosis induced by DNA lesions mainly results from double-strand breaks and stalled replication forks, which activates DNA damage checkpoints networks consisting of DNA damage sensors, signal transducers and effector pathways. The central sensors are composed of the PI3K-related kinases and include ataxia telangiectasia mutated (ATM), ataxia telangiectasia and Rad3-related (ATR) and DNA-dependent protein kinase (DNA-PK). Upon activation by DSBs and DNA replication blocks, PI3Ks indicated above activate specific substrates that mediate replication fork stability, cell cycle arrest, DNA repair and apoptosis.

The accumulating evidence implicated PI3Ks and AKT are tightly coregulated in both checkpoint response and DNA DSB repair. This reflected, predominantly, the regulatory role of AKT in NHEJ-mediated DNA repair. Indeed, AKT and DNA-PK were shown to activate each other to induce and maintain an effective NHEJ-mediated DNA repair. For example, Stronach et al. demonstrated that AKT relocates to the nucleus of cisplatin-resistant cancer cells where it is phosphorylated specifically on S473 by DNA-PK, and this activation inhibits cisplatin-mediated apoptosis. Importantly, direct interaction between DNA-PK and AKT in cisplatin-resistant but not sensitive cells was revealed by immunoprecipitation [25]. The regulatory role of DNA-PK in Akt-S473 phosphorylation was also shown for glioblastoma cells in response to IR [26]. The role of AKT in DNA-PKcs-dependent DNA DSB repair has been extensively studied by Toulany M. with co-authors. In particular, they showed that inhibition of AKT1 effectively sensitized non-small cell lung cancer cell lines to IR by inhibiting DNA-PKcs-dependent DNA DSB repair [27]. They also found that AKT (predominantly Akt1) mediated DNA-PKcs autophosphorylation at S2056 that is required for efficient DNA-DSB repair and release of DNA-PKcs from the DNA damage sites induced by IR. Akt1 played a critical role in formation of the functional complex to DNA duplex ends marked by Ku dimers [28]. Lastly, they found that Akt1 and Akt3, but not Akt2, interact with DNA-PKcs in K-Ras mutant cells and stimulate DSB repair, thereby protecting cancer cell against IR [29].

In contrast to NHEJ, the data illustrating the regulatory role of AKT in homology-mediated DNA repair (HR) is controversial. For example, Mueck K. with co-authors demonstrated that regulatory role of Akt1 in Rad51 foci formation in IR-treated NSCLC cells and recruitment of Rad51 recombinase to γ-H2AX foci, a well-known marker of DNA DSBs. This was in a concordance with a significant decrease of Rad51 protein in the nucleus of irradiated cells exhibiting AKT knockdown (AKT-KD). Lastly, the increased number of BRCA1 foci in AKT-KD cells exposed to IR also illustrated the impaired HR repair which was revealed by using SceI-based GFP-reporter assay [30]. In contrast, Plo I. with co-authors observed the opposite effect of AKT in homology-mediated DNA repair. For example, AKT silencing restored formation of IR-induced BRCA1 foci in breast cancer cells, whereas HR-related proteins (e.g., BRCA1 and Rad51) sequestered in the cytoplasm upon activation of AKT1. Important, this was observed in tumor cell lines and biopsies from AKT-high sporadic breast cancers, thereby illustrating that AKT1 inhibits homologous recombination in breast cancer cells in vitro and in vivo [31]. Similarly, negative regulatory role of Akt on homology-mediated repair was shown for BRCA1-deficient breast cancer cells. In particular, Akt1 promoted chromosome instability in Brca1-deficent cells by impairing nuclear localization of Chk1 and disrupting its interaction with Rad51, thereby leading to attenuation of homology-mediated repair [32].

Activation of AKT-mediated pathway is well-documented for the soft tissue sarcomas and can be used as independent prognostic factor for tumor recurrence, overall survival (OS) and disease-free survival (DFS) [12,33,34]. Similarly, activation of PI3-kinase/AKT pathway was well-documented for GIST cell lines and patient samples and played an important role for was critical to survival in IM-resistant GIST [1,2,3,7], thereby illustrating a rationale for combination therapy (e.g., of imatinib mesylate and AKT inhibitor) for patients with GIST [8].

Despite the sensitivity of STS and (in less extent) GIST to DNA-damaging agents was shown in multiple reports [20,21,22,23], however, to date, a little is known about the role of AKT in DNA DSB repair (in particular HR) in STS and GIST. Given that inhibition of FGF-signaling in GIST effectively sensitized them to Topo II inhibitors via attenuating HR-mediated DNA DSB repair [35], we sought to examine the downstream signaling pathways responsible for this phenomenon. We present here the novel data illustrating that AKT- but not a MEK-signaling pathway regulates an efficiency of homology-mediated DNA damage repair in STS and GIST. In particular, phosphorylated (i.e., activated) form of endogenous Akt physically interacts with Rad51 recombinase after DNA damage (Figure 8C). This was in consistency with previous data illustrating that Akt might be physically associated with various DNA DSBs repair proteins. For example, Toulany M. with the colleagues demonstrated that Akt1 phosphorylated at Ser472/Ser473 interacts with activated (i.e., phosphorylated at T2609) DNA-PKcs in various cell lines including NSCLC cells A549 and H460. Of note, this interaction was found shortly (3–5 min) of post IR exposure (4Gy), thereby illustrating an important regulatory role of Akt1 in DDR signaling [27]. The detailed analysis of this interaction demonstrated that Akt1 interacts with DNA-PKs through its C-terminal domain, stimulates autophosphorylation of DNA-PKcs at S2056, promotes its kinase activity and accumulation of DNA-PKcs at DNA-DSBs [28]. Similarly, by utilizing the proximity ligation assay, Sahlberg S. with co-authors demonstrated that phosphorylated forms of Akt and DNA-PKcs also interact with each other in IR-exposed colon cancer cell lines, whereas Akt deficiency significantly impaired the rejoining of radiation-induced DNA double strand breaks and sensitized colon cancer cells to IR [36]. Next, we found that AKT regulates Rad51 protein stability via proteasome-dependent pathway (Figure 8A,B) and MK-2206-induced inhibition of AKT-signaling has a strong impact of Rad51 expression, especially after Dox-induced DNA damage. Again, our data is in a close consistency with the data published recently by Mueck C. with co-authors illustrating that knockdown of Akt1 significantly reduced the amount of Rad51 protein in the nuclear fraction of irradiated non-small ling cancer cells [30]. We also observed that AKT signaling plays a regulatory role in the recruitment of Rad51 to DNA damage sites which was evidenced by a substantial decrease of number of residual Rad51 foci in Dox-treated tumor cells (Figure 7). As a result, inhibition of AKT-signaling pathway effectively sensitized STS and GIST to DNA-damaging agent, doxorubicin, and decreased tumor cell viability due by enhancing of apoptotic cell death (Figure 1 and Figure 2, Figures S3 and S4). 

Collectively, our data illustrates that overactivation of AKT-signaling pathway in STS and GIST might serve as a prospective molecular target to enhance cytotoxicity of DNA-topoisomerase II inhibitors against STS and GIST. This is in consistency with several reports illustrating a perspective role of PI3K/AKT/mTOR inhibitors to sensitize STS to standard chemotherapeutic regimens, including Topo II inhibitors. For example, Babichev Y. with colleagues demonstrated a high potency of PI3K vs. mTOR inhibitors used in combination of doxorubicin against LMS in vitro and in the leiomyosarcoma xenografts [37]. Similarly, AKT inhibition by pentacyclic triterpene ursolic acid (UA) effectively sensitized human STS cell lines in vitro to Dox treatment and induced their apoptosis [38]. Moreover, phosphorylation of AKT which was observed in STS cell lines after Dox treatment (10 M) illustrates that activation of AKT signaling represents a compensatory mechanism counteracting the cytotoxic and anti-proliferative effects of Dox [38]. This, in turn, illustrates a rationale of AKT inhibition in cancer cells treated with Dox. Our study effectively supplements these findings and illustrates the molecular mechanism responsible for this phenomenon. We show here for the first time that inhibition of AKT pathway in Dox-treated STS and GIST reduces Rad51 levels and inhibits recruitment of Rad51 recombinase to sites of DNA DSBs after Dox treatment, thereby attenuating the homology-mediated repair mechanisms. This was evidenced by substantial decrease of Rad51 foci that were co-localized with γ-H2AX when cancer cells were treated with Dox in presence of AKT inhibitor (Figure 7 and Figure S7). Moreover, data obtained from fractionation experiment revealed the substantially decrease in Rad51 level in chromatin fraction when cancer cells were treated with Dox in presence of MK-2206 but not with Dox alone (Figure S8). Thus, AKT inhibition may have a synergistic effect with Dox, standard chemotherapy for several types of STS due to inhibition of DNA DSB repair via attenuation of recruitment of Rad51 recombinase to the DNA damage lesions. Of note, we did not observe the enhanced phosphorylation of AKT in STS and GIST after Dox-induced DNA damage (Figure 2). This might be due to enhanced basal level of expression of phosphorylated form of AKT in STS and GIST cells and also in consistency with previous findings, illustrating that Dox treatment enhances AKT phosphorylation in sarcoma cell lines when was used in a high dose (10M), whereas no increase of AKT phosphorylation was observed when the cells were treated with 1M of Dox [38], which was similar to our experimental conditions. Of note, the effect of inhibition of AKT signaling might be not limited to sensitization of STS to the Topo II inhibitors. For example, MK-2206 and eribulin, a microtubule dynamics inhibitor, synergistically inhibited STS cell growth in vitro and in vivo, also revealing a rationale for the development of an AKT inhibitor in combination with eribulin for therapy of patients with STS [39]. Similarly to Dox-treated STS, expression of phosphorylated AKT significantly increased in eribulin-treated STS, thereby revealing AKT phosphorylation a common compensatory mechanism counteracting the cytotoxic and anti-proliferative effects of chemotherapeutic agents used for therapy of patients with STS. Thus, future studies with inhibitors targeting these pathways are warranted.

## 4. Materials and Methods

### 4.1. Chemical Compounds

Doxorubicin (Dox) were obtained from Sigma (St. Louis, MO, USA), MK-2206 and U0126 were purchased from Selleck Chem (Houston, TX, USA).

### 4.2. Antibodies

The following primary antibodies were used for immunoblotting and immunofluorescence: Cleaved form of caspase-3 (#9662S), phospho-AKT S473 (#4060P), AKT (#4691P), phospho-histone H2A.X Ser139 (#9718S) (Cell Signaling, Danvers, MA, USA), PARP (#436400) (Life Technologies, Carlsbad, CA, USA), γ-H2AX S139 (sc-517348), Rad51 (sc-8349) (Santa Cruz Biotechnology, Santa Cruz, CA, USA), Rad51 (ab-133534, Abcam, Burlingame, CA, USA), phosphor-RPA32 Ser4/8 (A300-245)(Bethyl Laboratories, Montgomery, TX, USA), beta-actin (A00730-200, Gene Script, Piscataway, NJ, USA), HRP-conjugated secondary antibodies for Western blotting were purchased from Santa Cruz Biotechnology (Santa Cruz, CA, USA) and Alexa Fluor 488- or Texas Red-conjugated secondary antibodies for immunofluorescence staining were obtained from Invitrogen (Carlsbad, CA, USA).

### 4.3. Cell lines and Culture Conditions

SK-LMS-1 leiomyosarcoma, RD rhabdomyosarcoma, HT-1080 fibrosarcoma and U2-OS osteosarcoma cell lines were purchased from American Type Culture Collection (ATCC). GIST T-1 cell line was generated from a metastatic pleural tumor from stomach GIST. This cell line exhibited the heterozygous 57-base pair deletion (V570-Y578) in *KIT* exon 11 [40]. IM-resistant GIST T-1R subline was generated in our laboratory. For this purpose, GIST T-1 cell line was continuously cultured with IM with a gradually increased concentration of the drug from 0.4 nM to 1000 Nm [41]. The IM-resistant GIST 430 cell line was generated from human GIST after developing resistance to IM therapy. This cell line has a heterozygous primary *KIT* exon 11 deletion (V560_L576del) and a secondary *KIT* exon 13-point mutation (V654A) [1]. GIST cell lines were cultured in a humidified atmosphere of 5% CO_2_ at 37 °C (LamSystems, Myass, Russia).

### 4.4. Cellular Survival MTS-Based Assay

STS and GIST cells were seeded in 96-well flat-bottomed plates (Corning Inc., Corning, NY, USA) and were cultured for 24 h. The cells were further incubated for 48–72 h with indicated concentrations of MK-2206, doxorubicin or DMSO (control). To assess the number of living cells, the MTS reagent (Promega, Madison, WI, USA) was added to the culture medium for at least 1 h. Cellular viability was determined using a MultiScan FC plate reader (Thermo Fisher Scientific, Waltham, MA, USA) at 492 nm. Calculated IC50 data were defined as the concentration of compound that inhibits cell growth by 50% at 48–72 h. Data were normalized to the DMSO-treated control group.

### 4.5. Western Blotting and Co-Immunoprecipitation (Co-IP)

For Western blotting analysis, cells were washed twice with PBS and lysed on ice for 20 min by using a RIPA buffer (25 mM Tris-HCl pH 7.6, 150 mM NaCl, 5 mM EDTA, 1% NP-40, 1% sodium deoxycholate and 0.1% SDS), supplemented with protease and phosphatase inhibitors. Cellular extracts were further centrifuged for 30 min at 13,000 rpm at 4°C. The pellet was removed, and the protein concentration in the supernatant (whole-cell lysate) was determined by the Bradford assay. The samples containing 30 μg of protein were resolved on 4% to 12% Bis-Tris or 3% to 8% Tris-acetate NuPAGE gels (Invitrogen, Carlsbad, CA, USA), transferred to a nitrocellulose membrane (Bio-Rad, Hercules, CA, USA), probed with specific antibody, and visualized by enhanced chemiluminescence (Western Lightning Plus-ECL reagent, Perkin Elmer, Waltham, MA, USA) on an gel-documenting system “Fusion Solo WL.2M” (Vilber Lourmat, Collégien, France). Densitometric analysis was performed in FIJI Software (Laboratory for Optical and Computational Instrumentation, University of Wisconsin, Madison, WI, USA).

For Co-IP, cells were washed with PBS and lysed on ice for 15 min using by TEB buffer (50 mM Tris-HCl pH 7.5, 150 mM NaCl, 1% NP-40 and 10% glycerol), supplemented with protease and phosphatase inhibitors. The lysates were cleared by centrifugation and further incubated with the corresponding precipitating Abs overnight (rotating device at 4 °C). Then protein A and G Sepharose beads (Santa Cruz Biotechnology, Santa Cruz, CA, USA) were added to the samples and incubated for 1 h (rotating device at 4 °C). The beads were washed 3 times with TEB buffer and resolved by SDS-PAGE. Then 1X sample buffer was added to the resolved precipitates and the mixture was boiled for 5 min. Subsequently, Western blotting was performed as indicated above.

### 4.6. Immunofluorescence Staining

Cells were seeded on glass coverslips coated with poly-L-lysine (Sigma-Aldrich, St. Louis, Missouri, USA) and allowed to attach for 48 h before treatment. After washing with PBS, cells were fixed in 4% paraformaldehyde solution (in PBS) for 30 min at 4 °C and further permeabilized with 0.5% Triton X-100 for 5 min at 4 °C. Then the cells were incubated for 30 min in blocking solution containing 10% normal goat serum and 0.5% bovine serum albumin (in PBS). The cells were further incubated for 30 min with the blocking solution containing 10% goat serum and 0.5% bovine serum albumin. After blocking procedure, the cells were incubated with primary antibodies for overnight at 4 °C. Next day the cells were washed twice with PBS, incubated with Alexa Fluor 488, or TexRed-conjugated secondary antibodies (Invitrogen, Carlsbad, CA, USA) for 30 min at room temperature in the dark. After brief DAPI (Sigma-Aldrich, St. Louis, MI, USA) staining, the coverslips were mounted on glass slides and visualized on fluorescence microscope “Olympus BX63” (Tokyo, Japan). Finally, the images were captured by using a Spot advanced imaging system.

### 4.7. RNA Extraction and Real-Time Quantitative PCR

Total RNA was extracted from cancer cells according to the standard protocol as described elsewhere [41]. PCR reaction mix for real-time quantitative PCR (qPCR) has consisted of the following components: 1 µL synthesized cDNA, 5x qPCRmix-HS SYBR (PB025, Evrogen, Moscow, Russia), 10 mM each forward and reverse PCR primers for *RAD51* or control genes. Real-time qPCR was carried according to the manufacturer’s protocol by the CFX96 Real-Time detection system (Bio-Rad, Hercules, CA, USA). The absolute levels of each mRNA were normalized relative to *GAPDH* as a control gene. The production of quantitative data based on the number of cycles required for the fluorescent detection amplifying of target genes (the Ct value). The relative level of expression of the target genes was based on the following formula 2^−ΔΔCt^.

### 4.8. Single-Cell Electrophoresis (Comet Assay)

Formation of DNA DSBs was assayed by using the alkaline version of single-cell electrophoresis. Comet slides were stained by SYBR Green (Trevigen, Gaithersburg, MD, USA) and visualized by fluorescent microscopy (Olympus BX63, Tokyo, Japan). Comet assay parameters (TM and OTM) were evaluated by the ImageJ software. 50 nuclei were evaluated per treatment. Differences between control and treated cells in TM and OTM were analyzed by Kruskal–Wallis test followed by Dunn’s test with Benjamini–Hochberg adjustment in R software (R Foundation for Statistical Computing, Vienna, Austria; URL https://www.R-project.org/).

### 4.9. Sub-Cellular Fractionation

For sub-cellular fractionation experiment cancer cells were lysed according to the protocol by Mladenov et al. [42]. Briefly, cells were collected, washed in PBS and Triton X-100-soluble fraction was extracted by using CSK-buffer. The pellet was washed with buffer A and then extracted with buffer A containing Benzonase^®^ Nuclease (Sigma, St. Louis, MO, USA). The supernatant was considered as a DNase-soluble fraction. The pellet was washed and extracted with buffers containing increasing concentrations of ammonium sulfate. The remaining pellet was labeled as the nuclear matrix fraction.

### 4.10. Images Quantification and Registration

Cells were seeded into 6-well plates and allowed to grow for 48 h before treatment. The cells were fixed, permeabilized and stained for γ-H2AX or Rad51 by using specific antibodies. Plates were imaged with a × 10 objective using a Cytell Cell Imaging System (GE Healthcare, Chicago, Illinois, USA). MyBioApp Protocol with specified parameters was created to acquire the data and to quantify the signal intensity in nuclei, cytoplasm, and in whole cells. DAPI (blue channel) was used for the nuclear masks, whereas Alexa Fluor 647 Mouse (red channel) was used for γ-H2AX-staining and Alexa Fluor 647 Rabbit (red channel) for Rad51-staining. Graphics illustrating the average intensity of the nuclear γ-H2AX-staining and Rad51-staining were automatically generated by using MyBioApp Protocol and then passed to MS Excel for further processing and analysis.

### 4.11. Statistics

All the experiments were performed for a minimum of three times. Data for each group are indicated as the mean ± standard deviation (SD). The results were considered statistically significant at *p* < 0.05. CompuSyn Version 1.0 (CompuSyn Inc., New York, NY, USA), based on the Chou–Talalay algorithm [43] was used to calculate the values of CI at each molar ratio of Dox and MK-2206. The combinations of the drugs indicated above that yielded CI values < 1 were considered as synergistic [44,45]. The degree of combination effects were also quantified by using R-package of the computational tool SynergyFinder (https://bioconductor.org/packages/release/bioc/html/synergyfinder.html) [46]. Zero potency interaction (ZIP) reference model was used to calculate synergy. Average delta score over a dose–response matrix was used as a summary interaction score for a drug combination. Value of delta score >0, =0 or <0 corresponds to the synergy, zero interaction and antagonism, respectively [46]. Means of normalized protein levels were compared by using the analysis of variance (ANOVA) with subsequent pairwise comparisons (Tukey HSD test) in R software (R Foundation for Statistical Computing, Vienna, Austria; URL https://www.R-project.org/).

## 5. Conclusions

Collectively, our data illustrates that inhibition of AKT-signaling pathway STS and GIST might be considered as a potent tool to enhance their sensitivity to doxorubicin, a topoisomerase II inhibitor. This might be due to the attenuation of homology-mediated DNA repair in AKT-inhibited tumor cells. In particular, we observed the decrease of Rad51 on the protein level in AKT-inhibited cells and attenuated recruitment of Rad51 recombinase to the chromatin and the sites of DSBs after Dox treatment. As a consequence of attenuation of homology-mediated repair of DNA DSBs, AKT-inhibited tumor cells underwent the apoptotic cell death after Dox treatment, which was evidenced by increases expression of well-known apoptotic markers—cleaved forms of caspase-3 and PARP.

## Figures and Tables

**Table 1:** IC50 values for Dox in STS and GIST treated in absence or presence of MK-2206, an AKT inhibitor.

IC50	MK-2206 (µM)	Dox (µM)	Dox + MK-2206 (µM)	Fold Increase
GIST T1-R	32.9 ± 4.3	0.77 ± 0.13	0.42 ± 0.1	1.8
GIST 430	17 ± 0.2	1.21 ± 0.08	0.59 ± 0.07	2.1
SK-LMS-1	13.5 ± 1.02	0.49 ± 0.05	0.3 ± 0.03	1.6
RD	26.4 ± 1	0.79 ± 0.08	0.47 ± 0.03	1.7
U2-OS	27.8 ± 1.2	0.49 ± 0.07	0.22 ± 0.04	2.2
HT-1080	19.7 ± 0.4	0.116 ± 0.005	0.05 ± 0.006	2.3

**Table 2:** CI values for each molar ratio of Dox and MK-2206 in STS and GIST.

Cell Line	Molar Ratio of DOX: MK-2206	CI Value ± SD
RD	1:11.62	1.41 ± 0.23
	1:5.81	0.07 ± 0.01
	1:2.90	0.15 ± 0.02
	1:1.45	0.31 ± 0.04
	1.38:1	0.34 ± 0.04
SK-LMS-1	1:11.62	1.18 ± 0.10
	1:5.81	0.58 ± 0.06
	1:2.90	0.60 ± 0.02
	1:1.45	0.46 ± 0.05
	1.38:1	0.33 ± 0.03
HT-1080	1:11.62	0.32 ± 0.06
	1:5.81	0.35 ± 0.05
	1:2.90	0.41 ± 0.06
	1:1.45	0.83 ± 0.09
	1.38:1	1.68 ± 0.60
U2-OS	1:11.62	0.19 ± 0.02
	1:5.81	0.30 ± 0.02
	1:2.90	0.26 ± 0.01
	1:1.45	0.26 ± 0.02
	1.38:1	0.40 ± 0.10
GIST T1-R	1:11.62	0.45 ± 0.05
	1:5.81	0.60 ± 0.06
	1:2.90	0.42 ± 0.05
	1:1.45	0.64 ± 0.04
	1.38:1	0.57 ± 0.06
GIST 430	1:11.62	2.32 ± 0.42
	1:5.81	0.43 ± 0.07
	1:2.90	0.61 ± 0.09
	1:1.45	0.69 ± 0.11
	1.38:1	0.75 ± 0.11

## Supplementary Materials

Supplementary materials can be found at https://www.mdpi.com/1422-0067/21/22/8842/s1.
