# Supplementary material for: Inhibition of AKT-Signaling Sensitizes Soft Tissue Sarcomas (STS) and Gastrointestinal Stromal Tumors (GIST) to Doxorubicin via Targeting of Homology-Mediated DNA Repair
Source: Int J Mol Sci. 2020 Nov 22;21(22):8842. doi: 10.3390/ijms21228842 (PMC7700672; doi:10.3390/ijms21228842)
Supplement: Supplementary file 1 [file ijms-21-08842-s001.pdf]

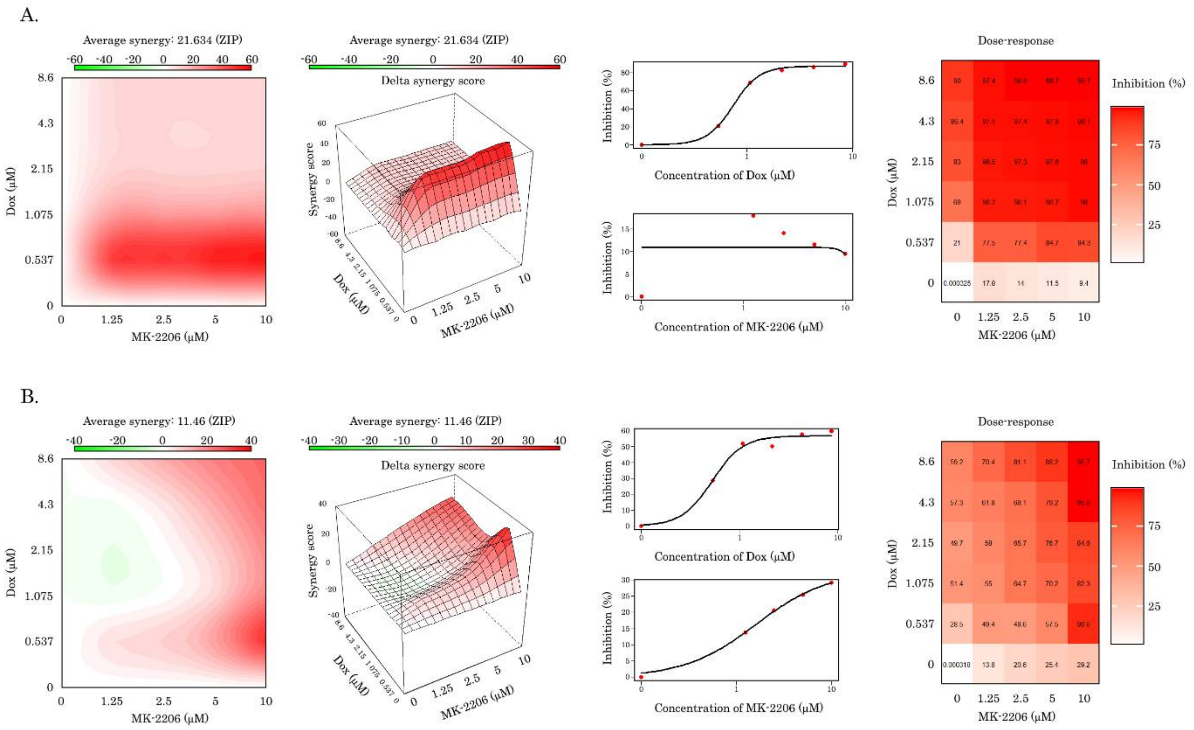

Suppl. Figure 1. *Synergy of Dox and AKT inhibitor in RD rhabdomyosarcoma and SK-LMS -1 leiomyosarcoma cells*. Dox combination with MK-2206 illustrates synergy against RD (A) and SK-LMS-1 (B). To determine the optimal synergy, the analysis was performed by using titrations of Dox and MK-2206. A surface plot of the Synergy score showed the most synergistic cell killing for RD rhabdomyosarcoma and SK-LMS -1 leiomyosarcoma cells at 0.537 μM Dox and 10 μM MK-2206. The average synergy for RD rhabdomyosarcoma cells was 21.634, whereas for SK-LMS -1 leiomyosarcoma was 11.460.

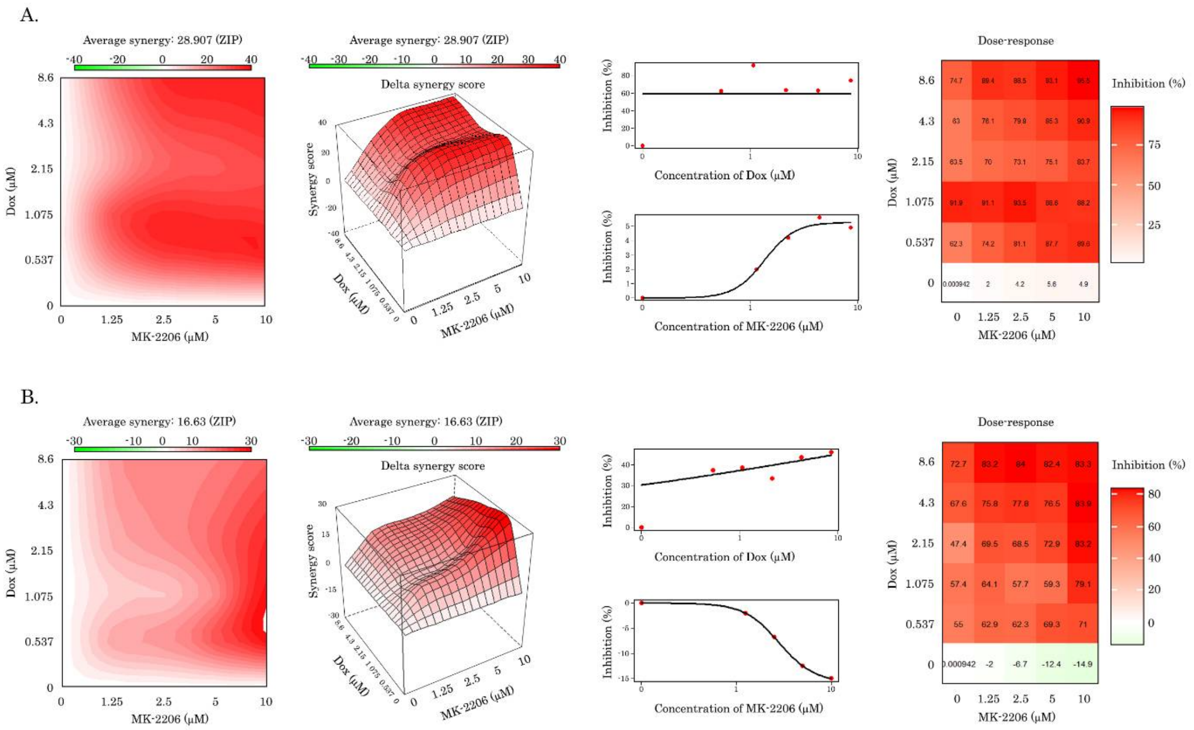

Suppl. Figure 2. *Synergy of Dox and AKT inhibitor in HT-1080 fibrosarcoma and U2-OS osteosarcoma cells.* Dox combination with MK-2206 illustrates synergy against HT-1080 (**A**) and U2-OS (**B**). To determine the optimal synergy, the analysis was performed by using titrations of Dox and MK-2206. A surface plot of the Synergy score showed the most synergistic cell killing for HT-1080 fibrosarcoma and U2-OS osteosarcoma cells at 0.537 µM Dox and 10 µM MK-2206. The average synergy for RD rhabdomyosarcoma cells was 28.907, whereas for SK-LMS -1 leiomyosarcoma was 16.630.

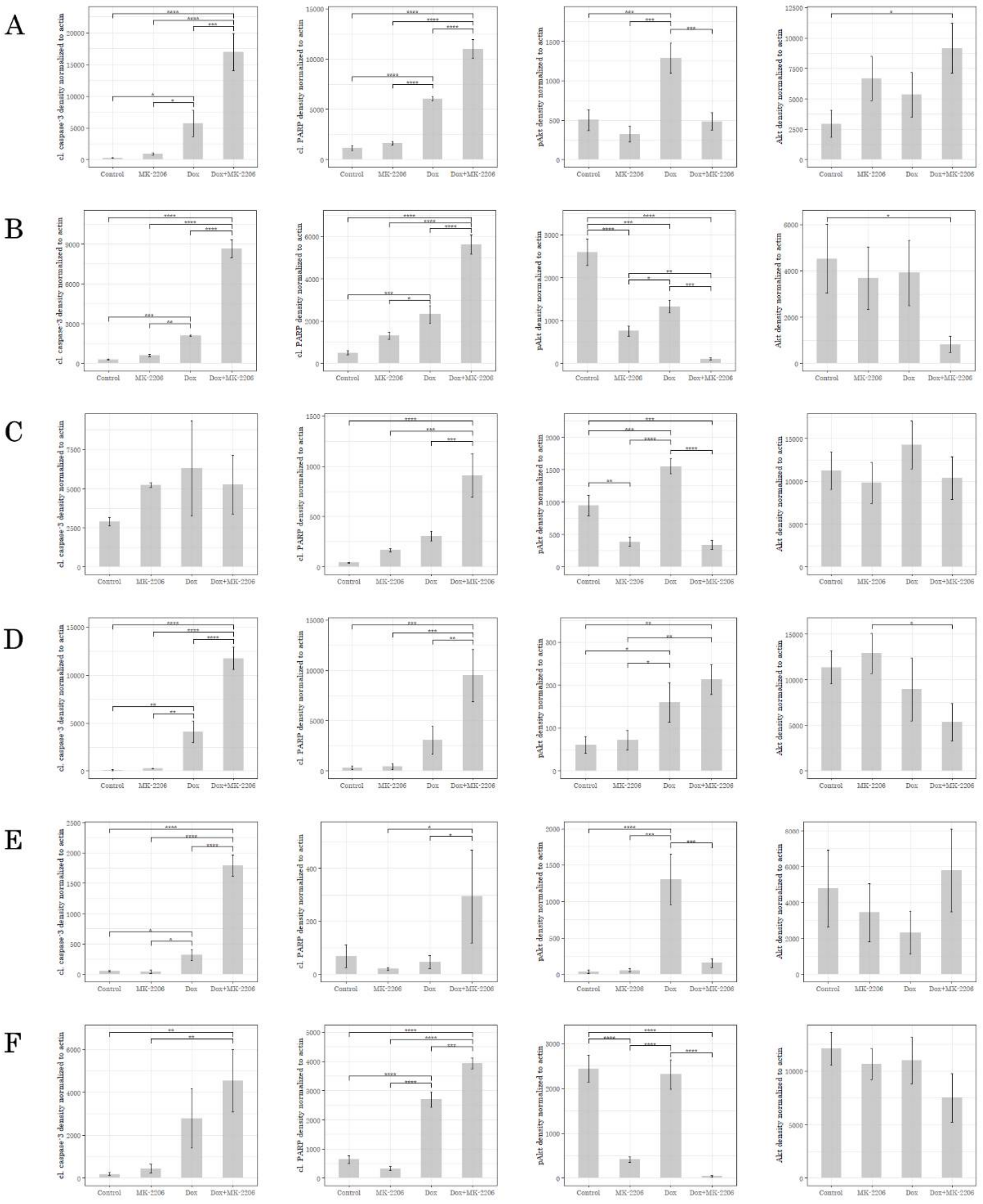

Suppl. Figure 3.1. Expression of apoptotic markers and AKT in cancer cells treated with Dox or MK-2006 alone, or in combination. Densitometric analysis of cleaved forms of caspase-3 and PARP, phosphorylated and total forms of Akt in RD rhabdomyosarcoma (**A**), U2-OS osteosarcoma (**B**), HT-1080 fibrosarcoma (**C**), GIST T-1R (**D**), GIST 430 (**E**) and SK-LMS-1 leiomyosarcoma (**F**) cell lines treated with DMSO (control), Dox (0.25 μg/ml), MK-2206 (5 μM) alone and in combination (e.g., Dox + MK-2206) for 48-72 h t; bars, SD. *- $p < 0.05$; ** - $p < 0.01$; *** - $p < 0.001$.

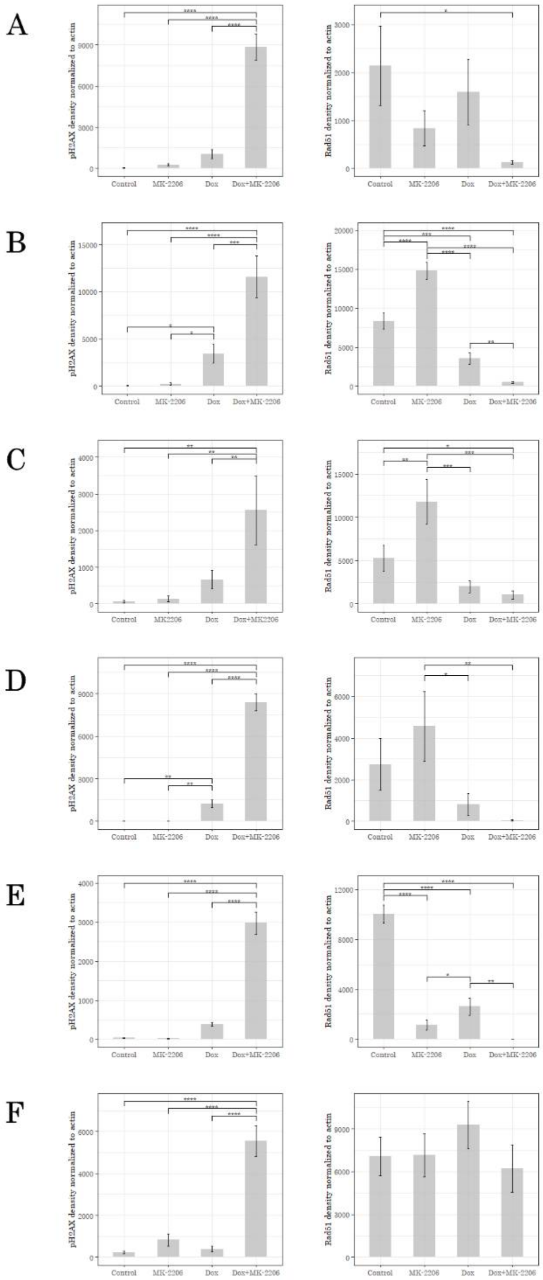

Suppl. Figure 3.2. Expression of γ-H2AX and Rad51 in cancer cells treated with Dox or MK-2006 alone, or in combination. Densitometric analysis of γ-H2AX and Rad51 in RD rhabdomyosarcoma (**A**), U2-OS osteosarcoma (**B**), HT-1080 fibrosarcoma (**C**), GIST T-1R (**D**), GIST 430 (**E**) and SK-LMS-1 leiomyosarcoma (**F**) cell lines treated with DMSO (control), Dox (0.25 μg/ml), MK-2206 (5 μM) alone and in combination (e.g., Dox + MK-2206) for 48-72 h; bars, SD.*- $p < 0.05$; ** - $p < 0.01$; *** - $p < 0.001$.

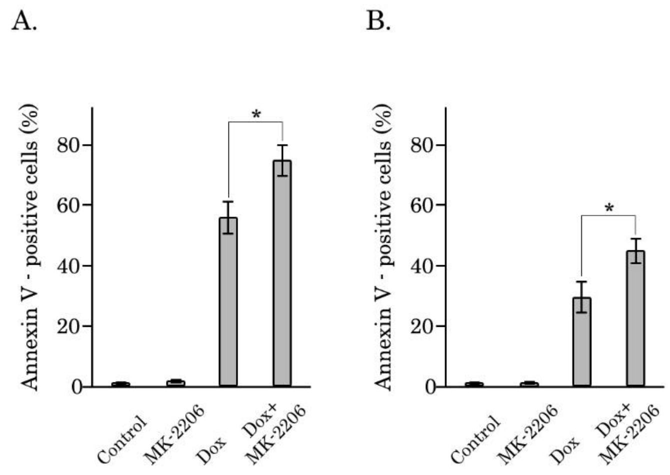

Suppl. Figure 4. Inhibition of AKT signaling increases the numbers of early- and late-apoptotic cells after Dox treatment. RD rhabdomyosarcoma (**A**) and GIST T-1R (**B**) were treated with DMSO (control), MK2206 (5μM), Dox (0.5 μM) or in combination. Cell were stained with Muse R Annexin V Dead cell kit (EMD Millipore Corp., Billerica, MA, USA) and analyzed by Muse Cell Analyzer. *- $p < 0.05$.

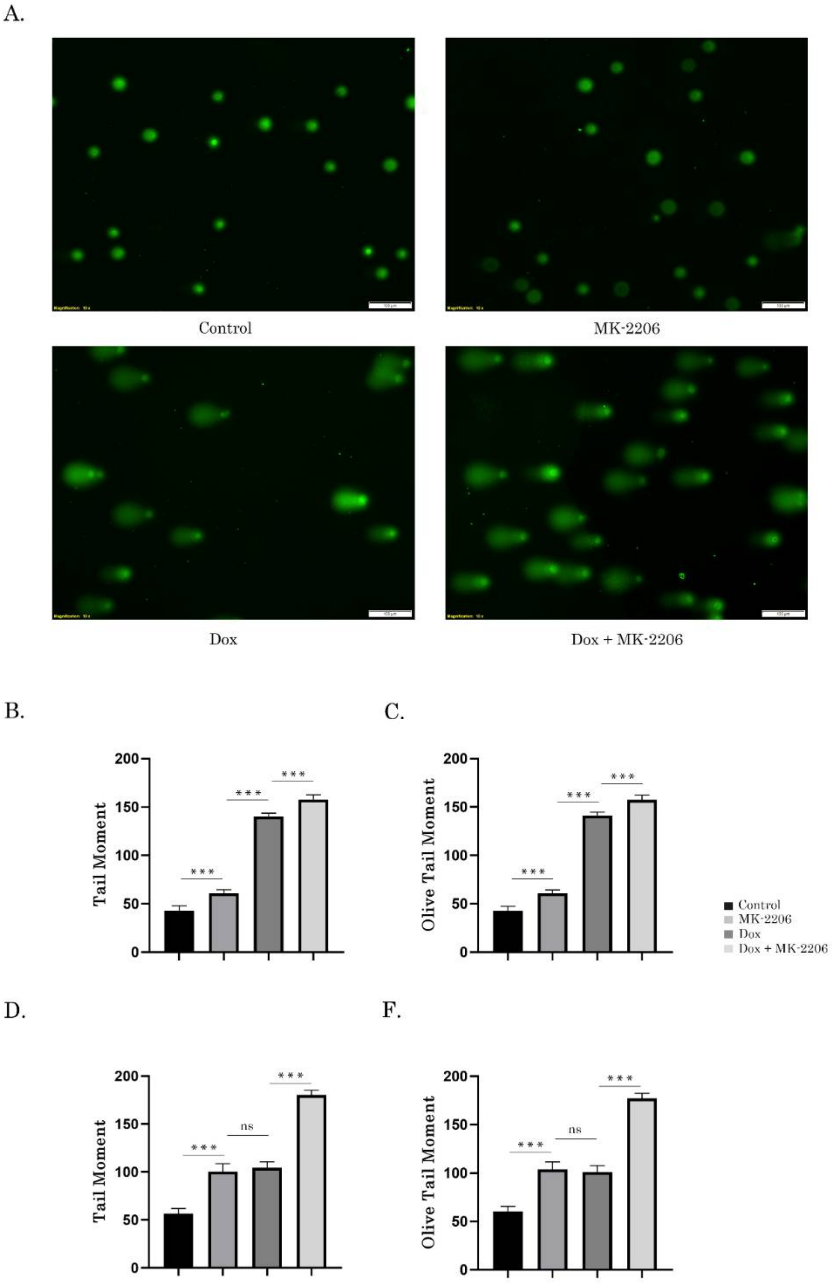

Suppl. Figure 5. *Inhibition of AKT-signaling attenuates DNA DSB repair in GIST.* GIST T-1R cells treated with DMSO (negative control) and doxorubicin (Dox) 0.25 µg/mL alone or in presence of MK-2206 (5 µM) for 48 h. (**A**) Representative images of comets from the experimental settings shown above (Scale bars = 100 µm). (**B, C**) Graphic depiction of the calculated Tail Moment (TM) and Olive Tail Moment (OTM) from alkaline Comet Assay shown in Figure A. (**D, F**) Graphic depiction of the calculated Tail Moment (TM) and Olive Tail Moment (OTM) from neutral Comet Assay in GIST T-1R cells treated with Dox and MK-2206 as shown above. Columns, mean of at least three independent experiments with a minimum of 100 cells counted per each experiment; bars, SE.*— $p < 0.05$; ** - $p < 0.01$; *** - $p < 0.001$.

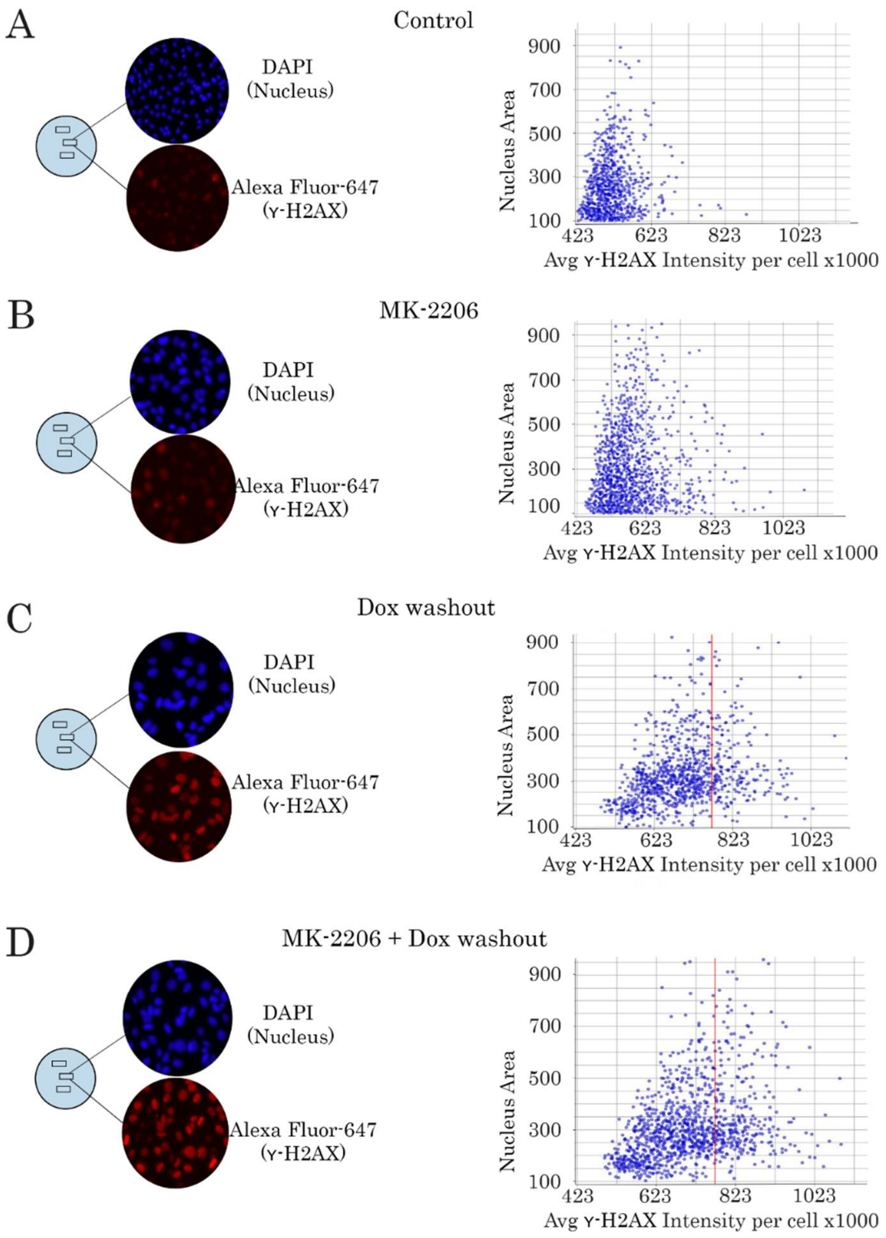

Suppl. Figure 6.1. Inhibition of AKT-signaling delays the kinetics of γ-H2AX decline in Dox-treated HT-1080 fibrosarcoma cells (representative experiment). Experiment design was similar as shown in Figure 4. *Left panel*–representative images of cancer cells treated with DMSO (**A**), MK-2206 (**B**), and doxorubicin (Dox) (**C**) or in presence with MK2206 after Dox washout (**D**). *Right panel* - Histograms illustrating the intensity of γ-H2AX-specific fluorescence at the single-nucleus level. Cells were stained with DAPI (blue) and γ-H2AX-specific antibody (red). The intensity of γ-H2AX-specific fluorescence was measured by using GE Cytell Cell Imaging System.

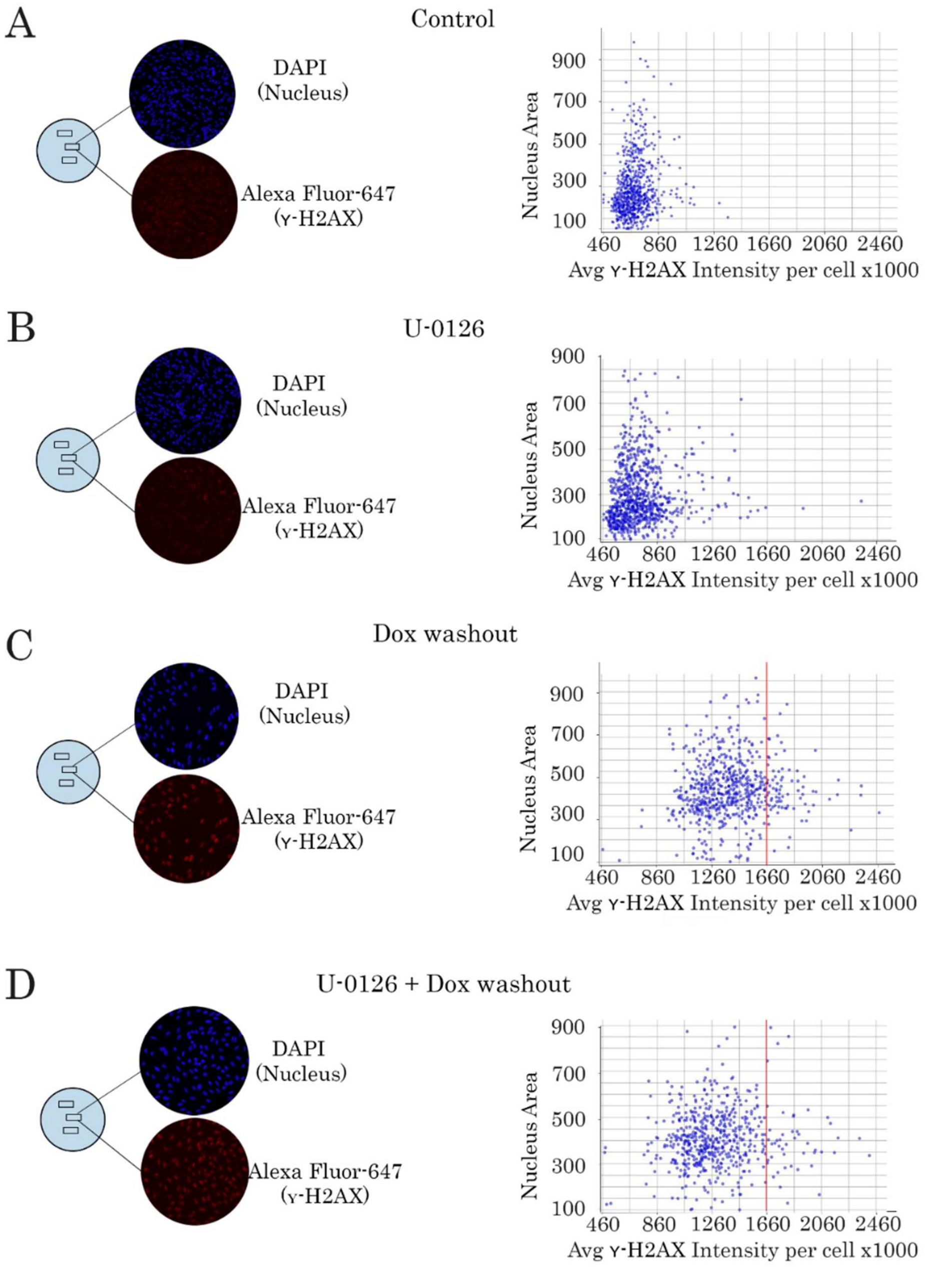

Suppl. Figure 6.2. Inhibition of MAPK-signaling has no impact on γ-H2AX kinetics in Dox-treated GIST T-1R cells (representative experiment). Experiment design was similar as shown in Figure 4. Left panel – representative images of cancer cells treated with DMSO **(A)**, U0126 (**B**), and doxorubicin (Dox) **(C),** or in presence of U0126 after Dox washout **(D).** Right panel - Histograms illustrating the intensity of γ-H2AX-specific fluorescence at the single-nucleus level. Cells were stained with DAPI (blue) and γ-H2AX-specific antibody (red) and intensity of γ-H2AX-specific fluorescence was measured as indicated above.

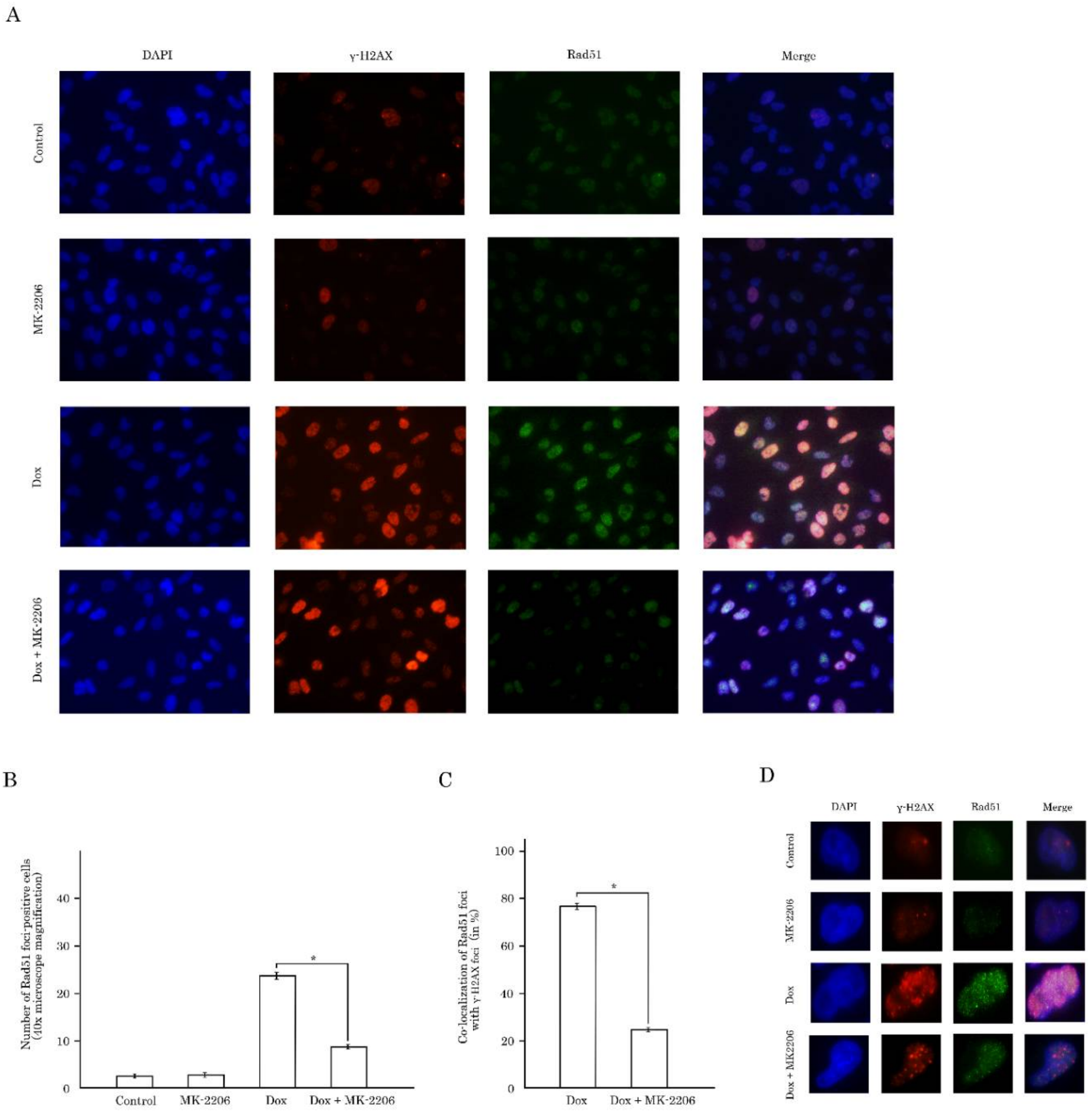

Suppl. Figure 7. Blockage of Akt signaling pathway disrupts involvement of the Rad51 protein in the repair of DNA DSBs in RD (rhabdomyosarcoma) cells. **(A)** Immunofluorescence staining of RD cells for γ-H2AX and Rad51. The cells were cultured in the presence of DMSO (control), MK-2206 (5 μM for 48 hours), doxorubicin (Dox) (0.25 μg/ml for 4 hours) or pretreated with MK-2206 (5 μM) for 48 hours prior Dox treatment. DAPI staining (blue) was used to outline the nucleus. Scale bars = 20 μm. **(B)** Graph depicting the number of RD cells positive for Rad51 foci after Dox treatment alone or in presence of MK-2206 from three independent experiments. Cells treated with DMSO (control) and MK-2206 were used as the negative controls. * $p < 0.05$. **(C)** Graph showing co-localization of Rad51 foci with γ-H2AX foci in RD cells after Dox treatment alone or in presence of MK-2206 from three independent experiments. * $p < 0.05$. **(D)** Distribution of γ-H2AX and Rad51 foci in the nucleus in a single-cell level. RD cells were pretreated with DMSO (control) or MK-2206 (5 μM) for 48 h prior Dox treatment (0.25 μg/mL for 4 hours). DAPI staining (blue) was used to outline the nucleus. Scale bars = 10 μm.

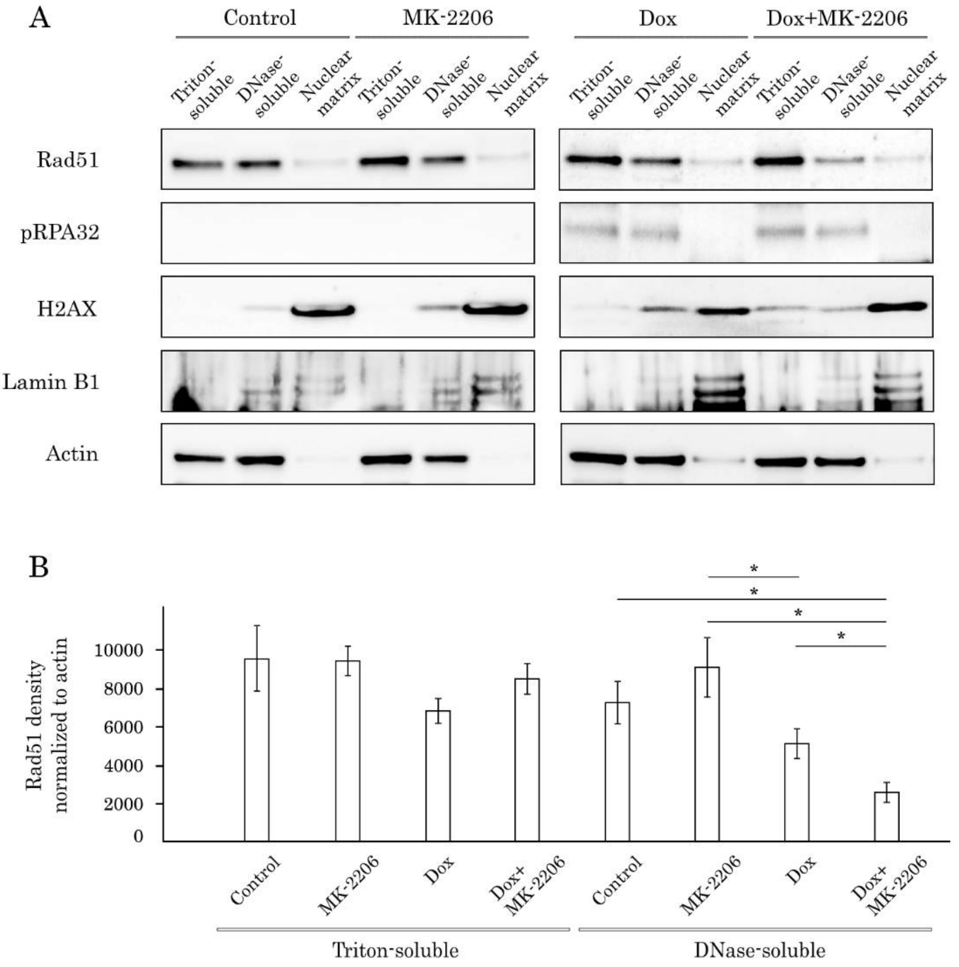

Suppl. Figure 8. Sub-cellular fractionation of Rad51 in GIST T-1R cells after treatment with MK-2206, Dox or in combination. (A) The cells were pretreated with DMSO or MK-2206 (10μM) for 24 h, treated with 1 μg/mL Dox for 16 h or left non-treated and further subjected to the biochemical fractionation into Triton X-100 soluble, DNase I soluble (chromatin) and nuclear matrix fractions. Equal amounts of protein from each fraction were subjected for immunoblotting and stained for Rad51, H2AX, Lamin B1. (B) Densitometric analysis of Rad51 distribution in the subcellular fractions indicated above; bars, SD.*- $p < 0.05$.

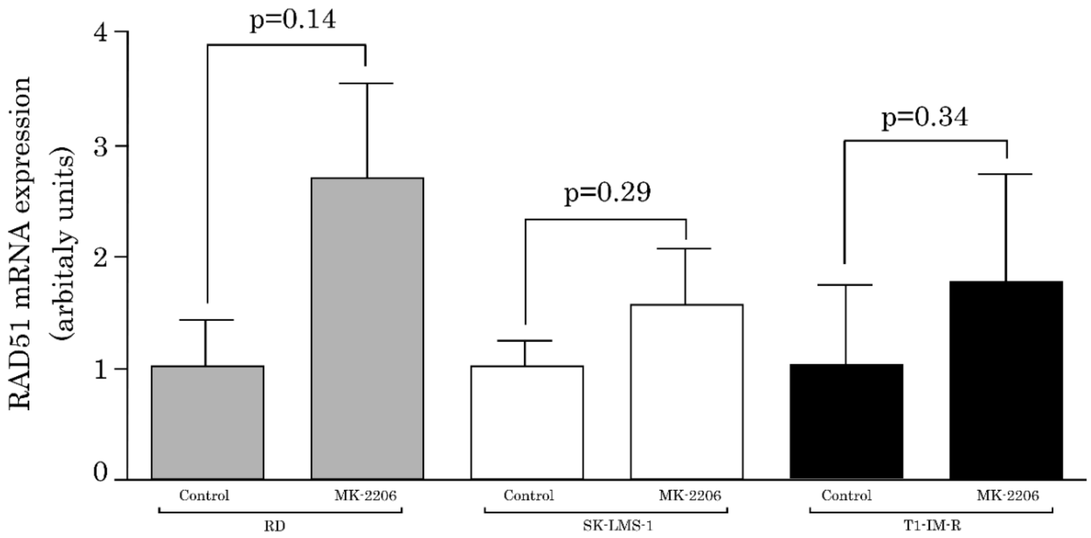

Suppl. Figure 9. Changes in the *RAD51* mRNA expression levels in RD rhabdomyosarcoma, SK-LMS-1 leiomyosarcoma and GIST T-1R cells treated with MK2206 (5 μmol/L) for 48 h or non-treated (control), as determined by quantitative RT-PCR. For internal control, the amplification of glyceraldehyde-3-phosphate dehydrogenase (*GAPDH*) was used.

Supplementary Figure Legends.

Suppl. Figure 1. *Synergy of Dox and AKT inhibitor in RD rhabdomyosarcoma and SK-LMS -1 leiomyosarcoma cells*. Dox combination with MK-2206 illustrates synergy against RD (A) and SK-LMS-1 (B). To determine the optimal synergy, the analysis was peformed by using titrations of Dox and MK-2206. A surface plot of the Synergy score showed the most synergistic cell killing for RD rhabdomyosarcoma and SK-LMS -1 leiomyosarcoma cells at 0.537 μM Dox and 10 μM MK-2206. The average synergy for RD rhabdomyosarcoma cells was 21.634, whereas for SK-LMS -1 leiomyosarcoma was 11.460.

Suppl. Figure 2. *Synergy of Dox and AKT inhibitor in HT-1080 fibrosarcoma and U2-OS osteosarcoma cells.* Dox combination with MK-2206 illustrates synergy against HT-1080 (A) and U2-OS (B). To determine the optimal synergy, the analysis was performed by using titrations of Dox and MK-2206. A surface plot of the Synergy score showed the most synergistic cell killing for HT-1080 fibrosarcoma and U2-OS osteosarcoma cells at 0.537 μM Dox and 10 μM MK-2206. The average synergy for RD rhabdomyosarcoma cells was 28.907, whereas for SK-LMS -1 leiomyosarcoma was 16.630.

Suppl. Figure 3.1*. Expression of apoptotic markers and AKT in cancer cells treated with Dox or MK-2006 alone, or in combination.* Densitometric analysis of cleaved forms of caspase-3 and PARP, phosphorylated and total forms of Akt in RD rhabdomyosarcoma (A), U2-OS osteosarcoma (B), HT-1080 fibrosarcoma (C), GIST T-1R (D), GIST 430 (E) and SK-LMS-1 leiomyosarcoma (F) cell lines treated with DMSO (control), Dox (0.25 μg/ml), MK-2206 (5 μM) alone and in combination (e.g., Dox + MK-2206) for 48-72 h t; bars, SD. *- $p < 0.05$; ** - $p < 0.01$; *** - $p < 0.001$.

Suppl. Figure 3.2. *Expression of γ-H2AX and Rad51 in cancer cells treated with Dox or MK-2006 alone, or in combination.* Densitometric analysis of γ-H2AX and Rad51 in RD rhabdomyosarcoma (A), U2-OS osteosarcoma (B), HT-1080 fibrosarcoma (C), GIST T-1R (D), GIST 430 (E) and SK-LMS-1 leiomyosarcoma (F) cell lines treated with DMSO (control), Dox (0.25 μg/ml), MK-2206 (5 μM) alone and in combination (e.g., Dox + MK-2206) for 48-72 h; bars, SD.*- $p < 0.05$; ** - $p < 0.01$; *** - $p < 0.001$.

Suppl. Figure 4. *Inhibition of AKT signaling increases the numbers of early- and late-apoptotic cells after Dox treatment.* RD rhabdomyosarcoma (A) and GIST T-1R (B) were treated with DMSO (control), MK2206 (5μM), Dox (0.5 μM) or in combination. Cell were stained with Muse R Annexin V Dead cell kit (EMD Millipore Corp., Billerica, MA, USA) and analyzed by Muse Cell Analyzer. *- $p < 0.05$.

Suppl. Figure 5. *Inhibition of AKT-signaling attenuates DNA DSB repair in GIST.* GIST T-1R cells treated with DMSO (negative control) and doxorubicin (Dox) 0.25 μg/mL alone or in presence of MK-2206 (5 μM) for 48 h. (A) Representative images of comets from the experimental settings shown above (Scale bars = 100 μm). (B, C) Graphic depiction of the calculated Tail Moment (TM) and Olive Tail Moment (OTM) from alkaline Comet Assay shown in Figure A. (D, F) Graphic depiction of the calculated Tail Moment (TM) and Olive Tail Moment (OTM) from neutral Comet Assay in GIST T-1R cells treated with Dox and MK-2206 as shown above. Columns, mean of at least three independent experiments with a minimum of 100 cells counted per each experiment; bars, SE.*— $p < 0.05$; ** - $p < 0.01$; *** - $p < 0.001$.

Suppl. Figure 6.1. Inhibition of AKT-signaling delays the kinetics of γ-H2AX decline in Dox-treated HT-1080 fibrosarcoma cells (representative experiment). Experiment design was similar as shown in Figure 4. *Left panel*–representative images of cancer cells treated with DMSO (A), MK-2206(B), and doxorubicin (Dox). *Right panel* - Histograms illustrating the intensity of γ-H2AX-specific fluorescence at the single-nucleus level. Cells were stained with DAPI (blue) and γ-H2AX-specific antibody (red). The intensity of γ-H2AX-specific fluorescence was measured by using GE Cytell imager as shown in "Materials and methods".

Suppl. Figure 6.2. Inhibition of MAPK-signaling has no impact on γ-H2AX kinetics in Dox-treated GIST T-1R cells (representative experiment). Experiment design was similar as shown in Figure 4. *Left panel* – representative images of cancer cells treated with DMSO (A), U0126 (B), and doxorubicin (Dox). *Right panel* - Histograms illustrating the intensity of γ-H2AX-specific fluorescence at the single-nucleus level. Cells were stained with DAPI (blue) and γ-H2AX-specific antibody (red) and intensity of γ-H2AX-specific fluorescence was measured as indicated above.

Suppl. Figure 7. *Blockage of Akt signaling pathway disrupts involvement of the Rad51 protein in the repair of DNA DSBs in RD (rhabdomyosarcoma) cells.* (A) Immunofluorescence staining of RD cells for γ-H2AX and Rad51. The cells were cultured in the presence of DMSO (control), MK-2206 (5 μM for 48 hours), doxorubicin (Dox) (0.25 μg/ml for 4 hours) or pretreated with MK-2206 (5 μM) for 48 hours prior Dox treatment. DAPI staining (blue) was used to outline the nucleus. Scale bars = 20 μm. (B) Graph depicting the number of RD cells positive for Rad51 foci after Dox treatment alone or in presence of MK-2206 from three independent experiments. Cells treated with DMSO (control) and MK-2206 were used as the negative controls. * $p < 0.05$. (C) Graph showing co-localization of Rad51 foci with γ-H2AX foci in RD cells after Dox treatment alone or in presence of MK-2206 from three independent experiments. * $p < 0.05$. (D) Distribution of γ-H2AX and Rad51 foci in the nucleus in a single-cell level. RD cells were pretreated with DMSO (control) or MK-2206 (5 μM) for 48 h prior Dox treatment (0.25 μg/mL for 4 hours). DAPI staining (blue) was used to outline the nucleus. Scale bars = 10 μm.

Suppl. Figure 8. *Sub-cellular fractionation of Rad51 in GIST T-1R cells after treatment with MK-2206, Dox or in combination.* (A) The cells were pretreated with DMSO or MK-2206 (10μM) for 24 h, treated with 1 μg/mL Dox for 16 h or left non-treated and further subjected to the biochemical fractionation into Triton X-100 soluble, DNase I soluble (chromatin) and nuclear matrix fractions. Equal amounts of protein from each fraction were subjected for immunoblotting and stained for Rad51, H2AX, Lamin B1. (B) Densitometric analysis of Rad51 distribution in the subcellular fractions indicated above; bars, SD.*- $p < 0.05$.

Suppl. Figure 9. Changes in the *RAD51* mRNA expression levels in RD rhabdomyosarcoma, SK-LMS-1 leiomyosarcoma and GIST T-1R cells treated with MK2206 (5 μmol/L) for 48 h or non-treated (control), as determined by quantitative RT-PCR. For internal control, the amplification of glyceraldehyde-3-phosphate dehydrogenase (*GAPDH*) was used.
